# Machine learning for accurate detection of small airway dysfunction-related respiratory changes: an observational study

**DOI:** 10.1186/s12931-024-02911-1

**Published:** 2024-07-24

**Authors:** Wen-Jing Xu, Wen-Yi Shang, Jia-Ming Feng, Xin-Yue Song, Liang-Yuan Li, Xin-Peng Xie, Yan-Mei Wang, Bin-Miao Liang

**Affiliations:** 1 Department of Respiratory and Critical Care Medicine, West China School of Medicine and West China Hospital, Sichuan University, No. 37 Guoxue Alley, Chengdu, 610041 China; 2 Machine Intelligence Laboratory, College of Computer Science, Sichuan University, Chengdu, 610065 China; 3 West China School of Medicine, West China Hospital, Sichuan University, Chengdu, Sichuan P. R. China; 4 College of Electrical Engineering and Automation, Sichuan University, Chengdu, 610065 China; 5 Institute of Traditional Chinese Medicine of Sichuan Academy of Chinese Medicine Sciences(Sichuan Second Hospital of T.C.M), Chengdu, 610000 China

**Keywords:** Machine learning, Impulse oscillometry, Small airway dysfunction, Preserved pulmonary function

## Abstract

**Background:**

The use of machine learning(ML) methods would improve the diagnosis of small airway dysfunction(SAD) in subjects with chronic respiratory symptoms and preserved pulmonary function(PPF). This paper evaluated the performance of several ML algorithms associated with the impulse oscillometry(IOS) analysis to aid in the diagnostic of respiratory changes in SAD. We also find out the best configuration for this task.

**Methods:**

IOS and spirometry were measured in 280 subjects, including a healthy control group (*n* = 78), a group with normal spirometry (*n* = 158) and a group with abnormal spirometry (*n* = 44). Various supervised machine learning (ML) algorithms and feature selection strategies were examined, such as Support Vector Machines (SVM), Random Forests (RF), Adaptive Boosting (ADABOOST), Navie Bayesian (BAYES), and K-Nearest Neighbors (KNN).

**Results:**

The first experiment of this study demonstrated that the best oscillometric parameter (BOP) was R5, with an AUC value of 0.642, when comparing a healthy control group(CG) with patients in the group without lung volume-defined SAD(PPFN). The AUC value of BOP in the control group was 0.769 compared with patients with spirometry defined SAD(PPFA) in the PPF population. In the second experiment, the ML technique was used. In CGvsPPFN, RF and ADABOOST had the best diagnostic results (AUC = 0.914, 0.915), with significantly higher accuracy compared to BOP (*p* < 0.01). In CGvsPPFA, RF and ADABOOST had the best diagnostic results (AUC = 0.951, 0.971) and significantly higher diagnostic accuracy (*p* < 0.01). In the third, fourth and fifth experiments, different feature selection techniques allowed us to find the best IOS parameters (R5, (R5-R20)/R5 and Fres). The results demonstrate that the performance of ADABOOST remained essentially unaltered following the application of the feature selector, whereas the diagnostic accuracy of the remaining four classifiers (RF, SVM, BAYES, and KNN) is marginally enhanced.

**Conclusions:**

IOS combined with ML algorithms provide a new method for diagnosing SAD in subjects with chronic respiratory symptoms and PPF. The present study’s findings provide evidence that this combination may help in the early diagnosis of respiratory changes in these patients.

**Supplementary Information:**

The online version contains supplementary material available at 10.1186/s12931-024-02911-1.

## Background

Two major chronic respiratory disorders that can affect the small airways include asthma and chronic obstructive pulmonary disease (COPD). Evidence from prospective studies indicates that asthma and COPD may occur before small airway dysfunction (SAD) [1–3]. Symptoms of COPD and asthma include coughing, producing phlegm, dyspnea, and wheezing. The following symptoms may indicate SAD in some subjects: negative airway hyperresponsiveness (AHR) or bronchial reversibility (BR), which means the subject does not meet the pulmonary function criteria for COPD or asthma, and preserved pulmonary function (PPF, forced expiratory volume in 1 s (FEV1)/forced vital capacity (FVC) ratio ≥ 0.70 [4]). According to a large-scale multi-stage stratified sampling survey, about 40% of Chinese individuals 20 years of age and older have spirometrically characterized SAD [5]. Owing to the severe impact of SAD, it was crucial to identify and treat the condition early.

The “quiet zone” is made up of small airways (with an inner diameter of less than 2 mm), which had a huge cross-sectional area and contribute very little to the total airway resistance. [6] In clinical practice, spirometry was the most widely used technique to assess small-airway function. The parameters that were employed include FVC50% (FEF50%), FVC75% at expiration (FEF75%), and forced expiratory flow between 25% and 75% of FVC (FEF25–75%). At least two of the three small airway markers (FEF25–75%, FEF50%, and FEF75%) had a projected value of less than 65%, which was the definition of spirometry SAD [5]. However, spirometry requires good cooperation of subjects, and the great variability of values makes its reliability not universally accepted [7, 8]. An approach to measuring respiratory impedance based on the forced oscillation technique (FOT) is called impulse oscillometry (IOS). All that is needed for the IOS measurement is quiet tidal breathing, which is easy to do, appropriate for a broad spectrum of individuals, and yields a variety of respiratory physiological data. IOS is able to measure the respiratory mechanics during quiet tidal breathing, which sets it apart from spirometry. Because of externally overlaid oscillatory signals, it is independent of subject effort, unlike spirometry [9]. Furthermore, it appears to correlate better with small airway features and may be more sensitive in identifying SAD [10–12]. Since IOS can reflect the viscosity of the respiratory system through electrical resistance (RRS) and the elastic and inertial properties of the respiratory system through reactance (XRS), it can be combined with spirometry to gain more insight into individual pathological changes.

IOS was not currently frequently utilized in pulmonary function assessment, though. This approach’s drawback stems from the fact that it relied on electrical engineering ideas, which might be challenging to interpret in a clinical context. Another important consideration is the expensive inspection apparatus. Therefore, even though the IOS test is straightforward, a busy, inexperienced pulmonary function technician or primary care physician would find it challenging to interpret the resistance and reactance curves, as well as the derived values, without proper training and expertise. Furthermore, the analysis is challenging due to the findings for the IOS test values being dispersed. Consequently, machine learning (ML)-based computer-aided decision systems can enhance the functionality of IOS and support physicians in strengthening the diagnosis, monitoring, and treatment of chronic respiratory disorders, such as asthma and COPD.

In this context, we hypothesized that the use of ML methods in combination with IOS test would improve the diagnosis of small airway function in PPF populations. This study aims to evaluate the performance of several ML algorithms in diagnosing SAD in PPF population, and to find the best configuration.

## Materials and methods

### Study population

This was a single-centered, observational study in the Pulmonary Function Laboratory of West China Hospital, Sichuan University. Subjects were recruited and tested from May 1st to September 1st, 2020.

Included were adult patients undergoing pulmonary function tests as a result of persistent respiratory complaints. In addition, participants must meet the PPF requirements (FEV1/FVC ≥ 0.70) [4]. The following conditions had to be met in order to be excluded: restrictive pulmonary diseases (FVC < 80% predicted), asthma, interstitial lung diseases, lung cancer, respiratory infection within two weeks, myocardial ischemia, history of pulmonary surgery, and incomplete IOS due to tongue position errors, vocal cord closures, or swallowing. As healthy controls, we also enrolled never-smokers (those with ≤ 1 pack-year of tobacco smoking history) with a normal chest radiograph, no active pulmonary conditions, and no unstable cardiovascular disorders. Basic demographic data was gathered, such as height, weight, age, sex, and body mass index (BMI). Subjects received IOS, spirometry, and completed a questionnaire covering qualitative and quantitative evaluation of symptoms. Also, bronchial provocation tests or bronchodilator tests were performed to exclude asthma. The study was approved by the ethics committee of West China Hospital, Sichuan University, and all participants signed an informed consent before the procedure.

### Impulse oscillometry and parameters

In accordance with ERS guidelines, the respiratory resistance and reactance were measured using IOS equipment (MS-IOS Jaeger) [9]. Because forced expiration may alter airway tone, IOS was performed prior to spirometry [13]. Pressure oscillations generated by a loudspeaker were superimposed onto normal tidal breathing through a mouthpiece for 30 to 45 s, which ranged from 5 to 35 Hz in frequency. Sitting upright, subjects were asked to wear a nasal clip and exert manual compression on their faces to minimize the influence of cheek vibration and air leak.

The IOS parameters selected in this paper and their clinical significance are as follows:

(1) Respiratory resistance at 5 Hz (R5): reflects the total viscous resistance of the respiratory system, because it is mainly airway resistance, also known as total airway resistance.

(2) Respiratory resistance at 20 Hz (R20): reflects central airway resistance.

(3) The difference between R5 and R20 (R5–R20): reflects the frequency dependence of resistance, that is, peripheral airway resistance. That is, the change of respiratory system resistance when the oscillation frequency is gradually increased.

(4) (R5-R20)/R5(%): the ratio of peripheral airway resistance to total airway resistance.

(5) Reactance at 5 Hz (X5): reflects the total elastic resistance of the respiratory system. Because the elastic resistance of the lung and thorax is the main one, it is often called peripheral elastic resistance, and also includes gas compression in the airway and alveoli. X5 is generally negative, with higher negative values indicating greater elastic resistance.

(6) Reactance area (AX): The area enclosed by the Xrs f frequency curve between 5 Hz and Fres and the horizontal 0 axis. AX is the integration of the low frequency reactance.

(7) Resonant frequency (Fres): The inertial resistance and elastic resistance are in opposite directions. When the two are equal and cancel each other, the reactance of the respiratory system is zero.

### Spirometry and parameters

Spirometry was performed by a full MasterScreen PFT System (Jaeger Corp. Germany) according to the American Thoracic Society (ATS)/European Respiratory Society (ERS) guidelines [14]. FEV1, FVC, FEV1/ FVC, FEF25–75%, FEF50% and FEF75% were recorded as percentages of predicted values. The prediction equations are based on a large study of normal spirometry values in Chinese aged 4–80 years, which is recommended in the spirometry guideline in China [15].

### Data sets

The data collection used for the experiments included measurements from 280 participant groups. The data set contained information from the volunteers’ IOS test and lung function in addition to biological data like age, sex, height, and weight. The PPF patients without SAD (PPFN group) contributed 158 sets, the PPF patients with SAD (PPFA group) contributed 44 sets, and the healthy control group (CG group) contributed 78 sets. Using random sampling, the data set is split into training and test sets in a 7:3 ratio. All of the given results were from test sets. The adjustment of the hyperparameters was obtained by manual tuning, taking the hyperparameter with the best average result.

### The studied classifiers

The discrete data measured by IOS can be thoroughly analyzed by ML algorithms to identify potential relationships. These ML algorithms were assessed in this study based on the findings of earlier research and pre-experiments:

(1) Random forests: A method of decision tree analysis in which a supervised algorithm works through “bagging” approach to create multiple decision trees with a random subset of the data. These decision trees are then merged to get a more accurate and stable prediction [16]. 

(2) Support vector machine: A supervised ML algorithm that classifies data points by finding the optimal hyperplane that maximally separates different classes in a high-dimensional space [17]. 

(3) Naive Bayes: A probabilistic classifier based on Bayes’ theorem [18]. 

(4) Adaptive Boosting (ADABOOST): A statistical classification algorithm that is frequently used with other “weaker” ML algorithms (e.g., decision tree) to improve their performance. [19]

(5) K-Nearest Neighbor (KNN): A common unsupervised ML method, in which unsupervised algorithms aim to group input vectors into k clusters based on k averages of points (i.e., centroids) without referring to known, or labeled outcomes [20]. 

In addition, this study conducted feature selection and investigated the use of SelectKBest, RFECV, and SelectFromModel algorithms in this experiment in order to find IOS parameters with a better correlation with the experimental results and minimize the complexity of the experimental data set.

(6) SelectKBest : A feature selection method based on statistical tests, which selects K features that are most relevant to the target variable according to some evaluation index. [21]

(7) RFECV: A Feature selection method in scikit-learn that combines Recursive Feature Elimination (RFE) and Cross-Validation (CV) to select the best feature subset [22]. 

(8) SelectFromModel: A feature selection method in scikit-learn, which selects the most relevant features based on the feature importance of the supervised learning model. [23]

### Experiment design

This study involved the conduct of five experiments.

The first experiment’s goal was to assess each IOS parameter’s capacity to identify SAD in patients with PPF. The study’s criteria for diagnosing SAD were two out of the three small airway measurements (FEF25-75%, FEF50%, and FEF75%) having a predictive value of less than 65% according to spirometry. We examined two distinct scenarios: control versus PPF patients without SAD (CGvsPPFN) and control versus PPF patients with SAD (CGvsPPFA) in order to accurately assess the degree of airway blockage in patients with PPF. The two situations described were likewise assessed in the remaining studies.

The second experiment employed the ML algorithm and compared it to the results obtained using a single IOS parameter to ascertain whether the ML algorithm could achieve superior performance. The area under the ROC curve (AUC) was then selected as the performance evaluation metric. All IOS parameter characteristics for this experiment were included in the selection process.

In the third experiment, the effectiveness of SelectKBest as a feature selector for lowering complexity and determining the significance of various IOS parameters was evaluated. Five classifiers were used for training once SelectKBest had chosen the IOS parameters.

In the fourth and fifth experiments, two model-dependent feature selection algorithms were employed to investigate the significance of the 7 IOS feature parameters in this study.Recursive Feature Elimination with Cross-Validation, or RFECV, was used in Experiment 4. RFECV fits a machine learning model to data, ranks features according to their weights or importance, recursively removes the least important features, and uses cross-validation to assess model performance in each iteration. RFECV creates a performance curve by recording the results of varying numbers of features removed in each round. Using SelectFromModel, the most pertinent characteristics were chosen in Experiment 5 based on the significance of the features in a supervised learning model. To increase model efficiency and generalization while preserving important information, the technique selects features over a threshold, computes feature importance scores, trains a supervised learning model, and then generates a new feature set.

Hypothesis testing is necessary to contrast ML algorithms. A wide variety of parametric tests are available, often based on t-tests. The Wilcoxon Rank-Sum Test, the Kruskal-Wallis Test, and the Mann-Whitney U Test are a few of the most often used nonparametric tests [24–26]. We used the permutation test to do hypothesis testing of AUCs in this work. [27, 28].

## Results

Table 1 displays the individuals’ biological parameters, spirometry results, chronic respiratory complaints, and IOS data. There was no discernible difference between any of the three research groups’ biological characteristics. There was no discernible difference in symptoms between the groups with and without spirometer-defined SAD for individuals with persistent respiratory symptoms. PPFA patients exhibited considerably lower spirometry parameters (*p* < 0.05), as Table 1 illustrates.

**Table 1:** Characteristics of healthy controls and subjects with and without spirometry-SAD

	Control group *n* = 78	PPFN group *n* = 158	PPFA group *n* = 44	
Demographics				
Age(years)	38.33 ± 10.19	39.72 ± 12.83	42.57 ± 13.03	ns
BMI(kg/m^2^)	22.98 ± 2.54	22.13 ± 3.07	22.78 ± 3.21	ns
Sex: female/male	39/39	79/79	22/22	ns
Spirometry				
FEV1(%predicted)	107.25 ± 11.17	107.01 ± 11.98	95.57 ± 9.35	1.2−3−1
FVC (% predicted)	107.73 ± 12.98	106.03 ± 13.74	107.12 ± 11.05	ns
FEV1/FVC	84.57 ± 5.65	85.50 ± 5.86	75.08 ± 3.17	1.2−3−1
FEF25–75%(%predicted)	92.92 ± 21.46	93.22 ± 18.83	57.01 ± 6.33	1.2−3−1
FEF50% (% predicted)	100.61 ± 22.92	98.42 ± 18.88	60.55 ± 6.13	1.2−3−1
FEF75% (% predicted)	84.77 ± 28.32	87.37 ± 28.21	51.14 ± 10.28	1.2−3−1
IOS				
R5	0.28 ± 0.06	0.31 ± 0.06	0.34 ± 0.07	1−2−3−1
R20	0.26 ± 0.06	0.29 ± 0.05	0.31 ± 0.06	1−2.3−1
R5-R20	0.0191 ± 0.0231	0.0216 ± 0.0289	0.0348 ± 0.0319	1.2−3−1
R5-R20/R5	7.51 ± 6.51	7.43 ± 6.89	9.67 ± 7.94	ns
X5	-0.0977 ± 0.0277	-0.0984 ± 0.0275	-0.1059 ± 0.0322	ns
AXV	0.22 ± 0.13	0.26 ± 0.16	0.35 ± 0.20	1.2−3−1
Fres	10.46 ± 2.13	11.19 ± 2.57	12.78 ± 3.28	1.2−3−1
Chronic respiratory symptoms				
Cough, n (%)	/	124(78.5)	34(77.2)	
Sputum, n (%)	/	67(42.4)	20(45.4)	
Wheeze, n (%)	/	61(38.7)	18(40.9)	
Dyspnea, n (%)	/	48(30.4)	14(31.8)	

(The last column describes the comparisons between groups, in which the dot means non-significant change, while the dash means significant change.)

**Fig. 1:**
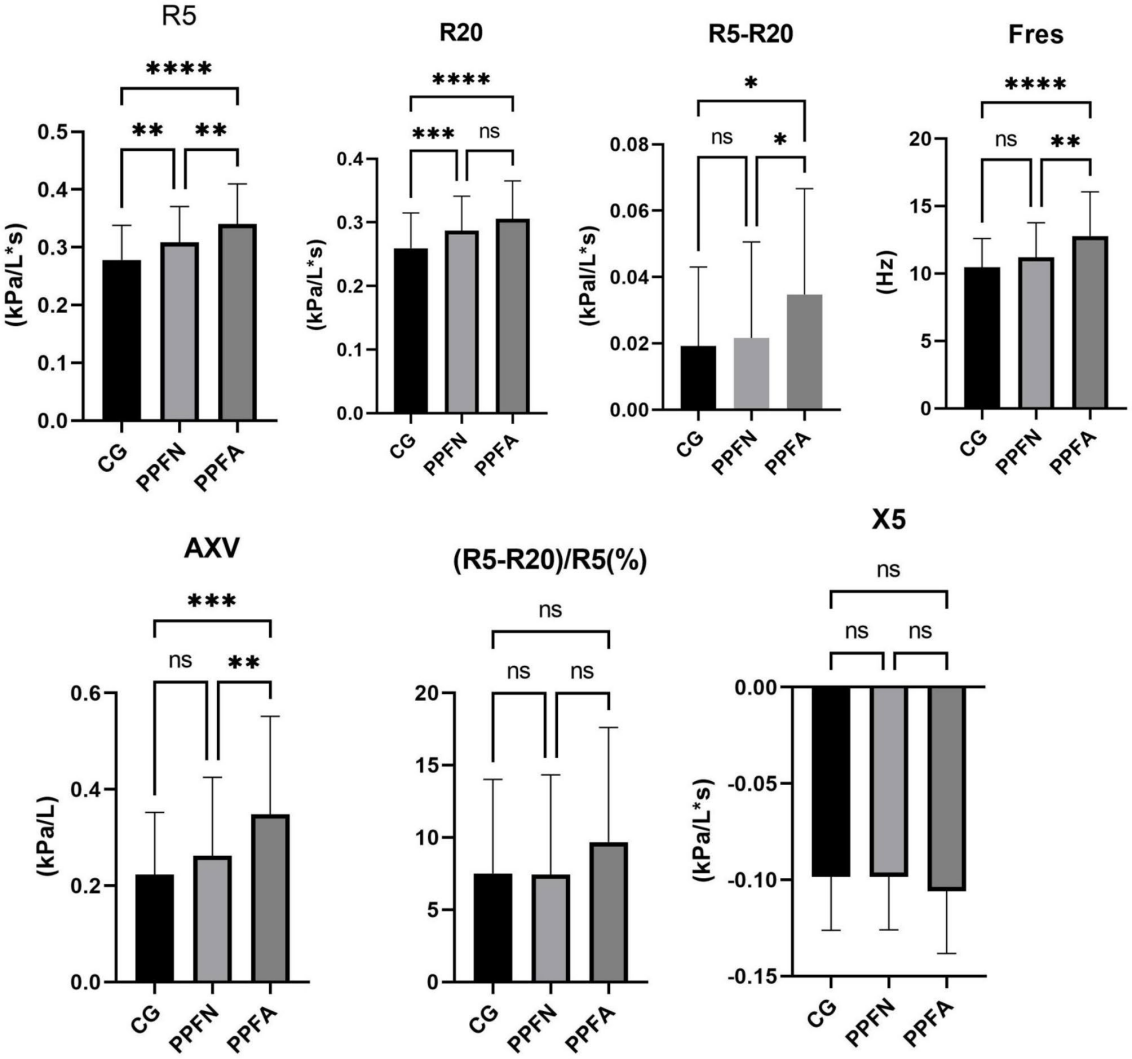
Comparison of IOS parameters among the three groups. Bar charts represented Mean + SD (M + SD). * indicates that there is a statistically significant difference comparing to each IOS parameter for each group. * *P* < 0.05, ** *P* < 0.01, *** *P* < 0.001, **** *P* < 0.0001

Figure 1’s bar graphs display the distinct features of the IOS parameters for the CG, PPFN, and PPFA groups. The majority of IOS parameters were substantially different (*p* < 0.05) across the three groups, according to the analysis of variance (ANOVA). PPF patients showed higher R5 and R20 when compared to healthy people. PPF patients consequently had greater airway resistance. In the meantime, patients with SAD in the PPF group showed greater values of R5, R5-R20, AXV, and Fres. The three groups’ R5-R20/R5 and X5 levels were comparable.

The first experiment: diagnostic accuracy of IOS parameters.

**Fig. 2:**
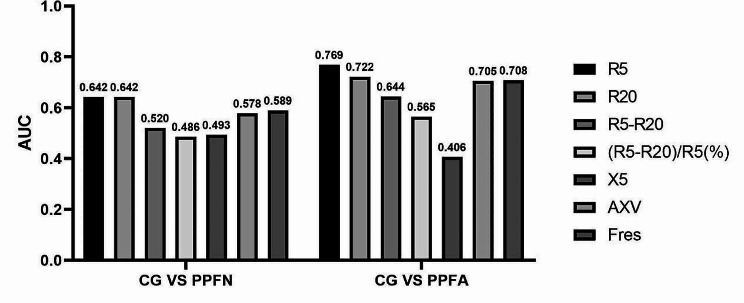
Results of experiment 1, describing the diagnostic accuracy of Impulse oscillometry in subjects with chronic respiratory symptoms and preserved pulmonary function. More detailed tables and graphs regarding these results are available in the Additional file.(Additional file Figure S1)

Figure 2 presents the findings from Experiment 1. As can be observed, R5 was the best IOS parameter (BOP) for PPF patient diagnosis, with moderate diagnostic accuracy (AUC = 0.642, AUC = 0.769) for CG vs. PPFN and CG vs. PPFA scenarios.

The second experiment of the study: diagnostic accuracy of the original IOS parameters associated with ML techniques.

**Fig. 3:**
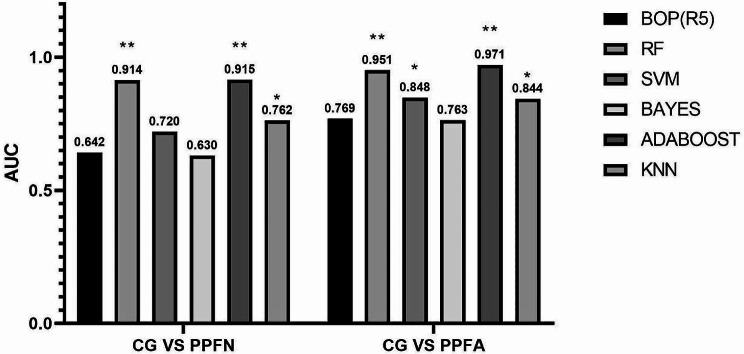
Results of experiment 2, describing the diagnostic accuracy of Impulse oscillometry with ML algorithms in subjects with chronic respiratory symptoms and preserved pulmonary function. Also, * indicates that there a statistically significant difference comparing to BOP (*p* < 0.05). * *P* < 0.05, ** *P* < 0.01. More detailed tables and graphs regarding these results are available in the Additional file.(Additional file Figure S2-S3)

Figure 3 presents the AUCs of the BOP, ML algorithm, and MIL classifier obtained in Experiment 2. It can be seen that the ML algorithm improves the AUC with high diagnostic accuracy in both cases, CGvsPPFN and CGvsPPFA. In the CGvsPPFN scenario, ADABOOST (AUC = 0.915) had the best performance, followed by RF (AUC = 0.914). Compared with BOP, RF, SVM, ADABOOST and KNN showed statistical differences. In the CGvsPPFA scenario, ADABOOST (AUC = 0.971) had the best performance, followed by RF (AUC = 0.951). Compared with BOP, RF, SVM, ADABOOST and KNN showed statistical differences.

The third experiment: diagnostic accuracy of the best original IOS parameters associated with ML techniques.

The IOS parameters used for the two cases, CGvsPPFN and CGvsPPFA, respectively, utilizing SelectKBest as the feature selector, are shown in Table 2.

**Fig. 4:**
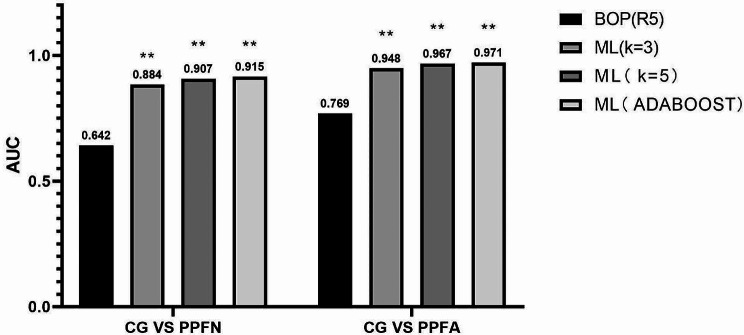
Summary of Experiment 2 and Experiment 3 (SelectKBest as a feature selector)—AUCs for the best oscillometric parameter (BOP), for the best ML algorithms in experiments 3, and the best ML algorithm with oscillometric parameters (ADABOOST). The figure indicates the best ML algorithm in each case. Also, * indicates that there a statistically significant difference comparing to BOP (*p* < 0.05). * *P* < 0.05, ** *P* < 0.01. More detailed tables and graphs regarding these results are available in the Additional file.(Additional file Figure S4-S7)

Experiments 2 and 3 had superior AUC outcomes, as shown by the data in Fig. 4. A similar pattern was seen in both cases when SelectKBest was used as the feature selector: as the number of features increased, the ML algorithm’s performance improved over time. When choosing 3/5 IOS feature parameters, the AUC value decreased slightly, but overall, the diagnostic performance was still better than BOP.

**Table 2:** The best IOS parameters in SelectKBest

	CG VS PPFN	CG VS PPFA
K = 3	R20 、(R5-R20)/R5 、Fres	R5、R20、Fres
K = 5	R5、R20、(R5-R20)/R5、AXV、Fres	R5、R20、(R5-R20)/R5、AXV、Fres

The fourth and fifth experiment: diagnostic accuracy of the IOS parameters associated with ML techniques.

**Fig. 5:**
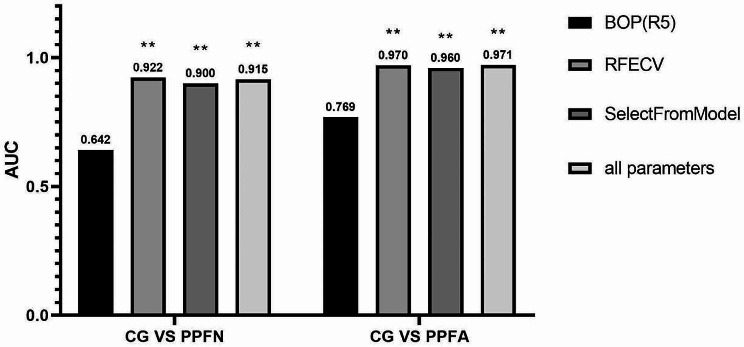
Summary of Experiment 4 and Experiment 5—AUCs for the best oscillometric parameter (BOP), for the best ML algorithms in experiments 4 and 5, and the best ML algorithm with oscillometric parameters. The figure indicates the best ML algorithm in each case. Also, * indicates that there a statistically significant difference comparing to BOP (*p* < 0.05). * *P* < 0.05, ** *P* < 0.01.More detailed tables and graphs regarding these results are available in the Additional file.(Additional file Figure S8-S19)

The best AUC findings for Experiments 4 and 5 are shown in Fig. 5. When compared to the full parameter, the IOS feature parameter’s diagnostic performance tends to be similar in both situations and to hold onto a high diagnostic value following feature selection.

The task configurations for each ML method classifier with the best performance across all experiments were summarized in Tables 3 and 4. In the two scenarios of CGvsPPFN and CGvsPPFA, among them, RF, SVM, ADABOOST, and KNN may increase the AUC, and the difference was statistically significant. Furthermore, The sensitivity, specificity, positive predictive value (PPV), and negative predictive value (NPV) of various individual ML classifiers are also reported.

**Table 3:** The best configuration for CG VS PPFN

	IOS parameters	AUC	Sensitivity (%)	Specificity (%)	NPV	PPV
BOP	R5	0.642	69.6	50.0	/	/
RF	R5、R20、(R5-R20)/R5、X5、AXV、Fres	0.922	79.1	89.7	0.6796	0.9398
SVM	R5、R20、R5-R20、X5、AXV	0.736	52.5	82.1	0.4604	0.8557
BAYES	R5、R20、(R5-R20)/R5、X5、AXV、Fres	0.642	77.9	46.2	0.5070	0.7455
ADABOOST	ALL	0.915	87.3	87.2	0.7727	0.9324
KNN	R5-R20、X5、AXV	0.811	53.8	89.7	0.4895	0.9140

**Table 4:** The best configuration for CG VS PPFA

	IOS parameters	AUC	Sensitivity (%)	Specificity (%)	NPV	PPV
BOP	R5	0.769	79.5	61.5	/	/
RF	ALL	0.951	86.4	92.3	0.9231	0.8636
SVM	R5、R20、(R5-R20)/R5、X5、AXV、Fres	0.853	72.7	89.7	0.8537	0.8000
BAYES	R5、R20、Fres	0.789	68.2	74.4	0.8056	0.6000
ADABOOST	ALL	0.971	88.7	97.4	0.9383	0.9512
KNN	R5、(R5-R20)/R5、Fres	0.845	70.5	80.8	0.8289	0.6739

## Discussion

For the purpose of early screening and treatment of respiratory disorders, a number of chronic respiratory disease guidelines, including GINA 2023 and GOLD 2024, advise early monitoring of changes in small airway function. In our previous study, we found that IOS is more sensitive to detect SAD than spirometry in subjects with chronic respiratory symptoms and PPF, and it correlates better with symptoms. IOS could be an additional method for SAD detection in the early stage of diseases [29]. Other similar research has demonstrated the usefulness of small airway function monitoring with IOS for clinical diagnosis [30–32]. We found only four correlated IOS parameters, including R5, R5-R20, AX, and Fres, which had low diagnostic efficacy, with none of the AUC values exceeding 0.7.

In order to facilitate the diagnosis of respiratory disorders, this study describes the design of a classifier for SAD diseases in the PPF population.By using machine learning approaches, this work aims to improve the diagnostic value of IOS for small airway dysfunction. Additionally, the best set of parameters and algorithms for this task was determined. Compared to a single IOS measure, the results show that this approach increases diagnostic accuracy and streamlines the clinical assessment of IOS.

Similar to our previous study, we found that R5 had the best AUC value, better sensitivity and slightly lower specificity among all parameters. After the introduction of the machine learning algorithm, the AUC, sensitivity, and specificity of the prediction model were very significantly improved.The best performance in both CGvsPPFN and CGvsPPFA scenarios was achieved by R5, which was the single IOS parameter used in the first experiment. The finding supports the presence of elevated airway resistance in patients with SAD, as measured by various methods including CT scans and bronchoscopy. It is important to note that these results are based on objective measurements rather than subjective evaluations [33, 34].

In the first case, it was more challenging to differentiate the control group from the patients with PPF who had preserved lung function. This was due to the small differences in IOS parameters. The AUC value was 0.642, indicating low diagnostic accuracy. In the second case, the increase in physiological abnormalities resulted in a greater difference in measured parameters, enabling R5 to easily distinguish between the two groups with an AUC of 0.769. These findings suggest that a single IOS parameter may not be sufficient to accurately identify the SAD situation in the PPF population.

The diagnostic accuracy was significantly enhanced through the utilization of RF, SVM, BAYES, ADABOOST, and KNN algorithms. It is clear that ADABOOST and RF produced the most favorable results followed by KNN, SVM and BYS.This breakthrough is mainly due to the use of ML algorithms.Similar to earlier research [35–38], feature selection permits the use of fewer characteristics without appreciably lowering performance. When SelectKBest was employed as a feature selector, the 3/5 relevant features were selected, respectively. Despite the final trend indicating that the results are superior when more parameters are used, the difference between using the least and most parameters is relatively minor. Furthermore, the results are superior when using the least parameters than when using BOP alone. This implies that feature selection can in fact result in good diagnostic value (AUC 0.948 and 0.967, respectively) with fewer IOS parameters. The most pertinent features are found through feature selection in both the CGvsPPFN and CGvsPPFA scenarios. Despite the fact that the approach only chose two sets of features, R20 and Fres had a significant intersection. This intersection is slightly different from the results of the ability of each single IOS parameter to diagnose SAD in patients with PPF, showing better diagnostic ability for R5 when using a single parameter. This suggests resonant frequency and central airway resistance, in addition to total airway resistance, have a significant role in the increased airway blockage observed in the PPF population.

Compared to the conventional classifier SelectFromModel, the RFECV method may produce superior results and has an efficient selection capability. While it does not increase the accuracy of diagnosis, it does display significant traits like R5, (R5-R20)/R5, and Fres. Feature selection was done to make the analysis easier to understand. We were able to discriminate between groups with clarity by using these three essential criteria. These results support the idea of a simple diagnostic model that can help explain the suggested medical decision support system’s findings and make it easier to apply in clinical settings.

Recent studies have shown that IOS is considered the most advanced technique for lung function analysis and is one of the most promising emerging techniques in the field [29, 39–41]. Despite its advantages in providing detailed and direct examination, IOS has not yet been widely used. However, because interpreting the metrics—which are based on electrical modeling—requires knowledge and experience, their application is restricted. This study shows how ML algorithms can improve the diagnosis of associated diseases and simplify the use of IOS, therefore improving healthcare for patients with SAD.

Early detection of abnormal respiratory changes in SAD can facilitate timely interventions that may limit disease progression, alleviate adverse symptoms, improve overall health, prevent complications and comorbidities, and reduce premature mortality [5, 42]. Since the 1980s, lung function analysis has been improved by artificial intelligence and machine learning techniques [43–48]. The present work expands on previous results by demonstrating that early aberrant respiratory alterations in SAD may be suggested by a combination of IOS measures and a clinical decision support system based on ML technology.

The algorithm presented in this work can be applied not just to SAD but to a variety of other conditions, including asthma, COPD, interstitial lung disease, and others. By establishing appropriate models and finding the best parameters, the relationship between physiological parameters and the development of the disease can be explored. This benefits the early screening of other respiratory diseases and the reduction of the disease burden on patients.

Clinical technology-wise, more thorough information can be obtained by combining IOS with other imaging modalities (such as MRI, CT, PET, etc.) and by developing real-time imaging technology and dynamic observation techniques. More information for clinical diagnosis and scientific study will be available with the improvement of image contrast and anatomical detail. [49] Concurrently, artificial intelligence and machine learning are integrated to analyse and interpret multiple data types, enhance the accuracy and credibility of clinical examination results, and develop automated and intelligent analysis tools. Encouraging data sharing and IOS standardization, creating a platform for data sharing and standardizing data formats, facilitating multi-center data comparison and analysis, and promoting the field’s progress are all crucial in the context of big data [50].

Finally, it is important to consider and clarify some significant limitations. Firstly, this study is limited to the Chinese population in a specific location. Therefore, it is not possible to ensure its generalisability to different populations. It is recommended that future studies investigate multi-centre data to expand the generalisability of the findings. The experimental design of this work followed globally recognised inclusion and exclusion criteria and was conducted in a typical clinical setting.

Additionally, it is important to note that the PPF population in China is relatively small due to low public health awareness. Many individuals do not seek medical attention promptly when experiencing clinical symptoms such as cough and chest tightness. Therefore, due to the relatively small size of the available dataset, it is necessary to carefully control the complexity of the ML model. In addition to the measures taken in this study to avoid overfitting, such as controlling hyperparameters, feature selection can also aid in controlling overfitting by reducing inputs. Another reason for using feature selection is that a smaller number of features can help simplify the analysis. Furthermore, utilising only three features enables the visualisation of group separation, aiding diagnostic interpretation.

## Conclusions

In this work, a variety of machine learning algorithms were utilized to create a clinical auxiliary diagnosis system that can identify respiratory anomalies in patients with PPF. In the initial disease stage (CGvsPPFN), respiratory oscillation parameters achieved low diagnostic accuracy (AUC = 0.642), but ML classifiers significantly improved accuracy (AUC ≥ 0.9). In the progressive disease stage (CGvsPPFA), using oscillation parameters alone yielded moderate accuracy (AUC = 0.769), while ML algorithms greatly enhanced accuracy (AUC ≥ 0.9). The developed diagnostic system simplifies IOS application in PPF patients, utilizing key IOS parameters identified through feature selection. All things considered, combining ML algorithms with IOS examination improves pulmonary function assessment in PPF patients, indicating future improvements in patient care.

### Electronic supplementary material

Below is the link to the electronic supplementary material.

Supplementary Material 1

## Data Availability

No datasets were generated or analysed during the current study.

## Abbreviations

COPD: Chronic obstructive pulmonary disease
SAD: Small airway dysfunction
PPF: Preserved pulmonary function
ML: Machine learning
RF: Random Forests
SVM: Support Vector Machines
BAYES: Navie Bayesian
ADABOOST: Adaptive Boosting
KNN: K-Nearest Neighbors
FEV1: The forced expiratory volume in 1st s
FVC: Forced vital capacity
AHR: Airway hyper-responsiveness
BR: Bronchial reversibility
FEF25–75%: The forced expiratory flow between 25 and 75% of FVC; FEF50%:The forced expiratory flow when 50% of FVC has been exhaled
FEF75%: The forced expiratory flow when 75% of FVC has been exhaled
IOS: Impulse oscillometry
Rrs: Resistance of the respiratory system
Xrs: Reactance of the respiratory system
R5: Respiratory resistance at 5 Hz
R20: Respiratory resistance at 20 Hz
R5–R20: The difference between R5 and R20
X5: Reactance at 5 Hz
Fres: Resonant Frequency
AX: Area under reactance curve between Fres and5 Hz
BOP: Best Oscillometric Parameter
ATS: American Thoracic Society
ERS: Europe Respiratory Society
ROC: Receiver Operator Characteristic
AUC: Area Under the Curve
PPV: Positive Predictive Value
NPV: Negative Predictive Value
BMI: Body Mass Index
