# Supplementary material for: Machine learning for accurate detection of small airway dysfunction-related respiratory changes: an observational study
Source: Respir Res. 2024 Jul 24;25:286. doi: 10.1186/s12931-024-02911-1 (PMC11270925; doi:10.1186/s12931-024-02911-1)

Figure S1 Results of experiment 1, describing the diagnostic accuracy of Impulse oscillometry in subjects with chronic respiratory symptoms and preserved pulmonary function.

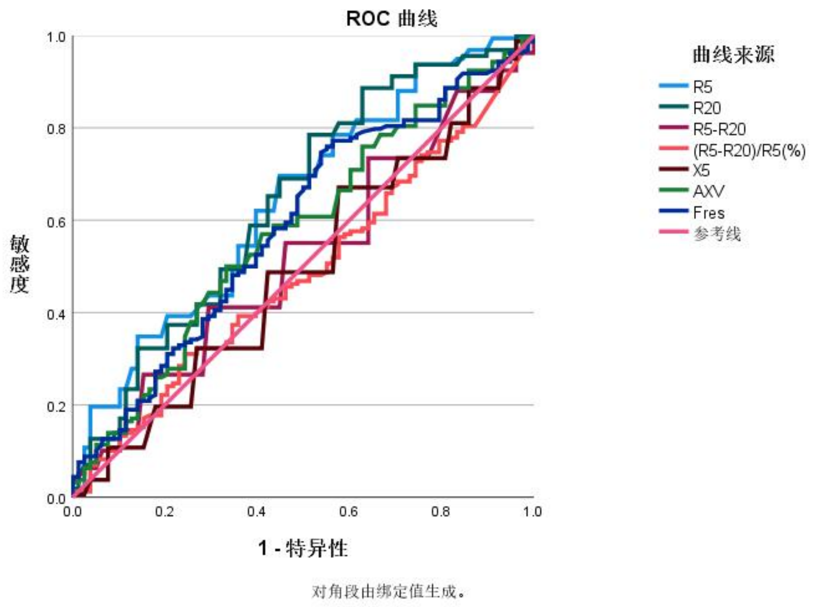

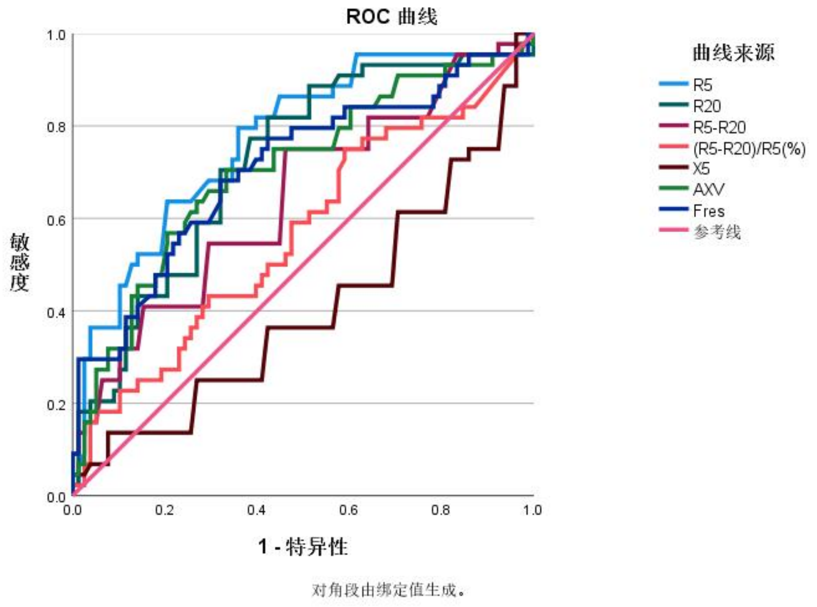

Figure S2 Results of experiment 2, CG vs PPFN, describing the diagnostic accuracy of Impulse oscillometry with ML algorithms in subjects with chronic respiratory symptoms and preserved pulmonary function.

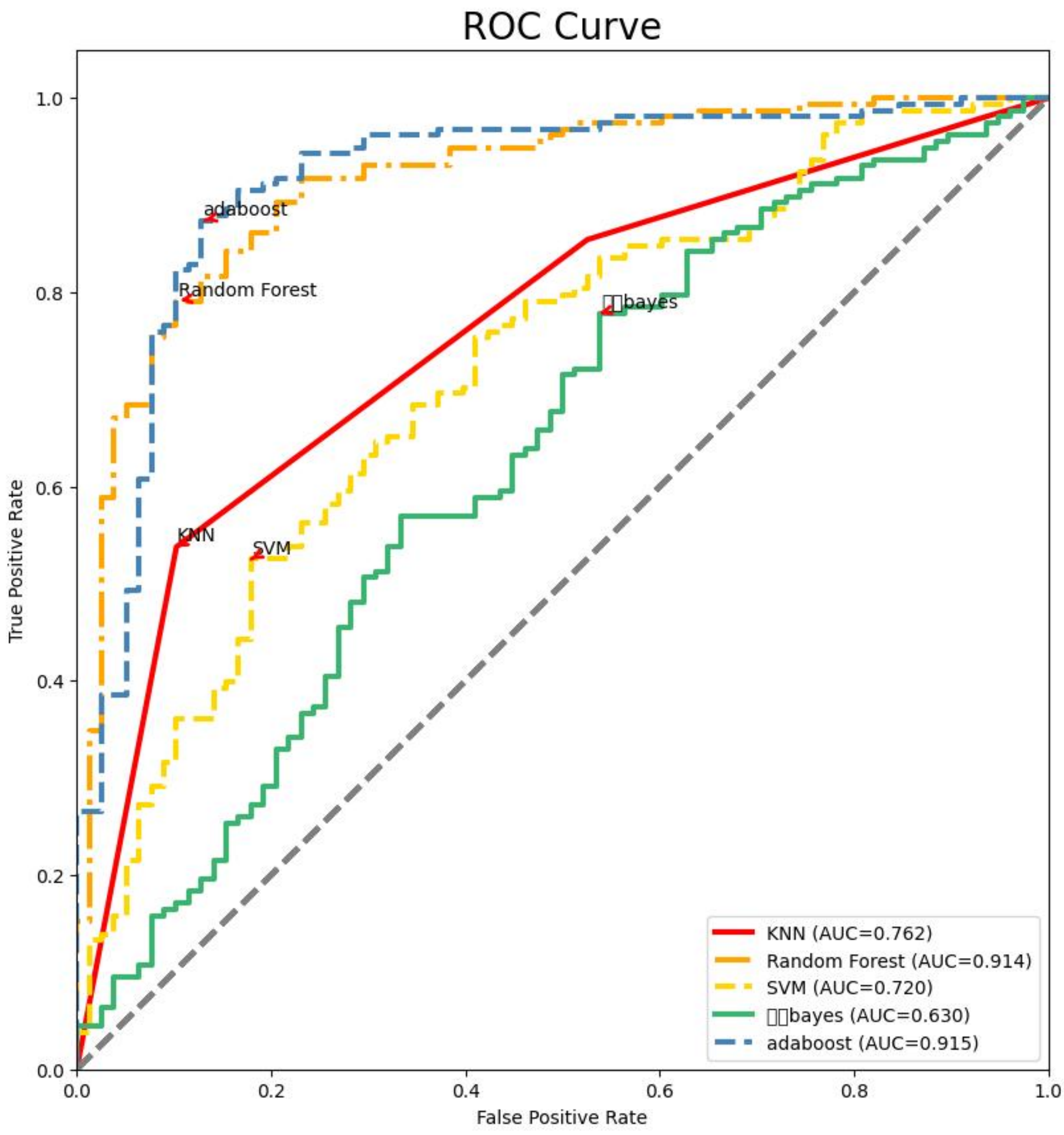

Figure S3 Results of experiment 2, CG vs PPFA, describing the diagnostic accuracy of Impulse oscillometry with ML algorithms in subjects with chronic respiratory symptoms and preserved pulmonary.

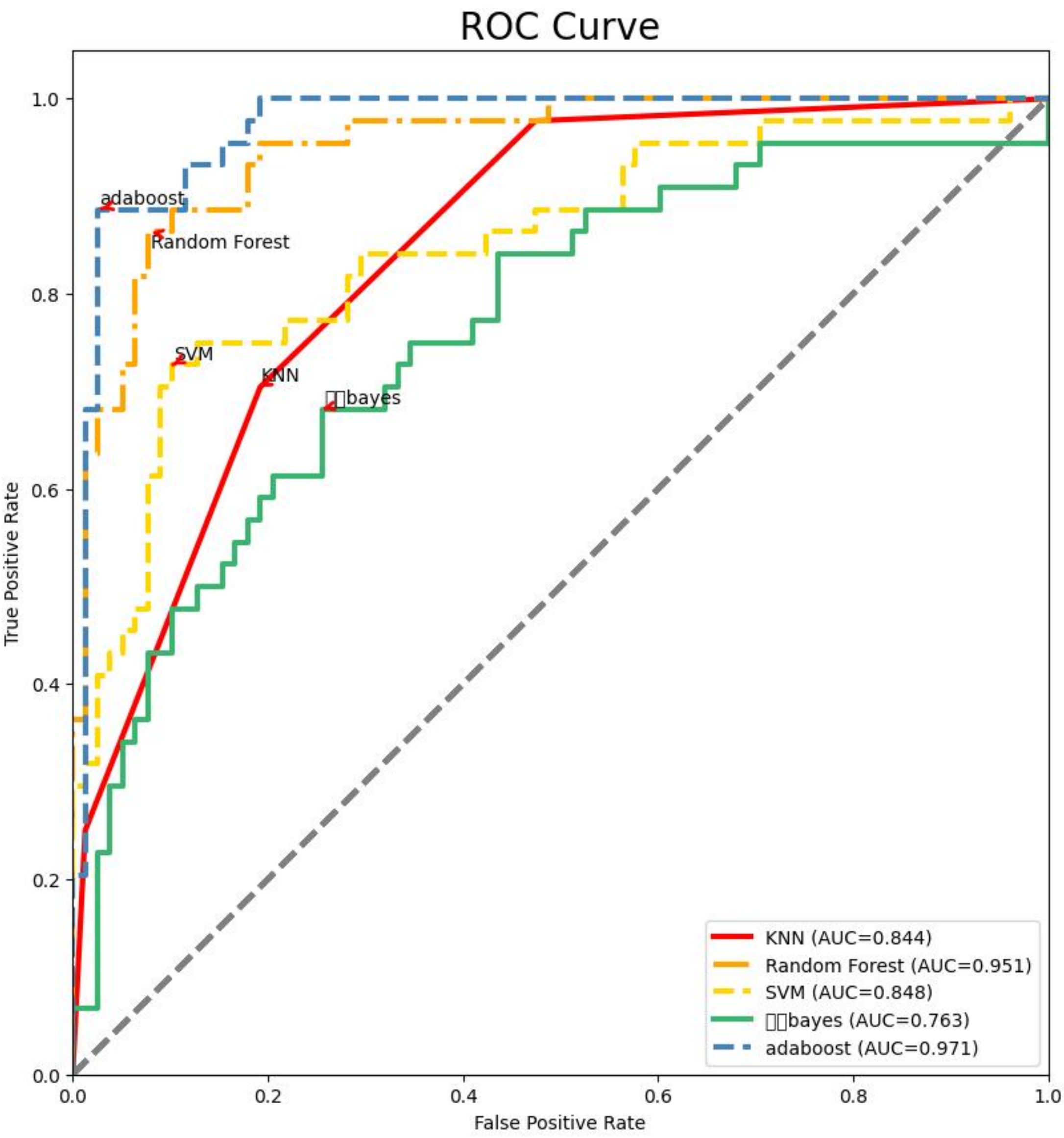

Figure S4 Results of experiment 3,K=3 (R20、(R5-R20)/R5、Fres), CG vs PPFN, describing the diagnostic accuracy of Impulse oscillometry with ML algorithms in subjects with chronic respiratory symptoms and preserved pulmonary.

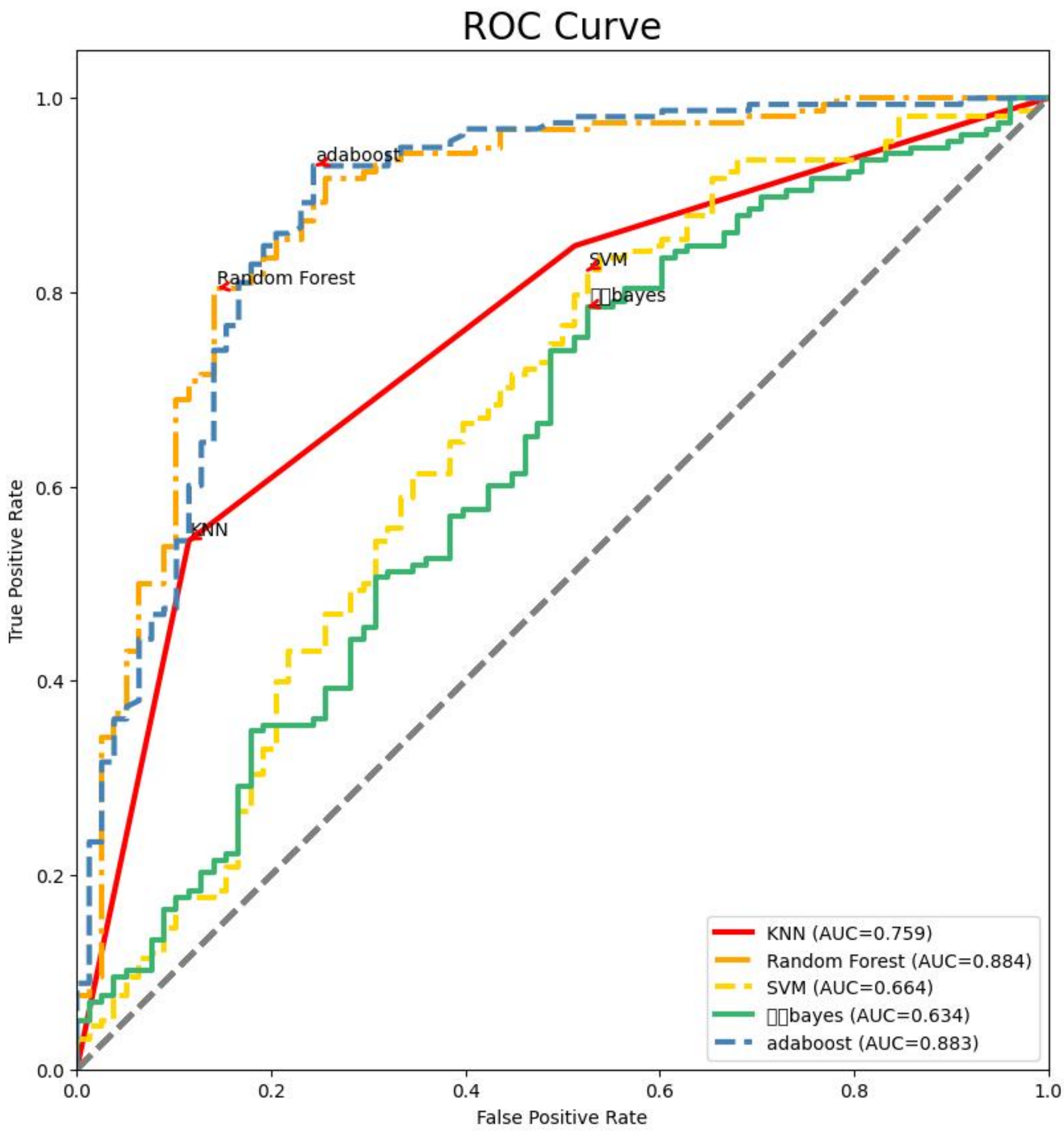

Figure S5　Results of experiment 3,K=3 (R5、R20、Fres), CG vs PPFA, describing the diagnostic accuracy of Impulse oscillometry with ML algorithms in subjects with chronic respiratory symptoms and preserved pulmonary.

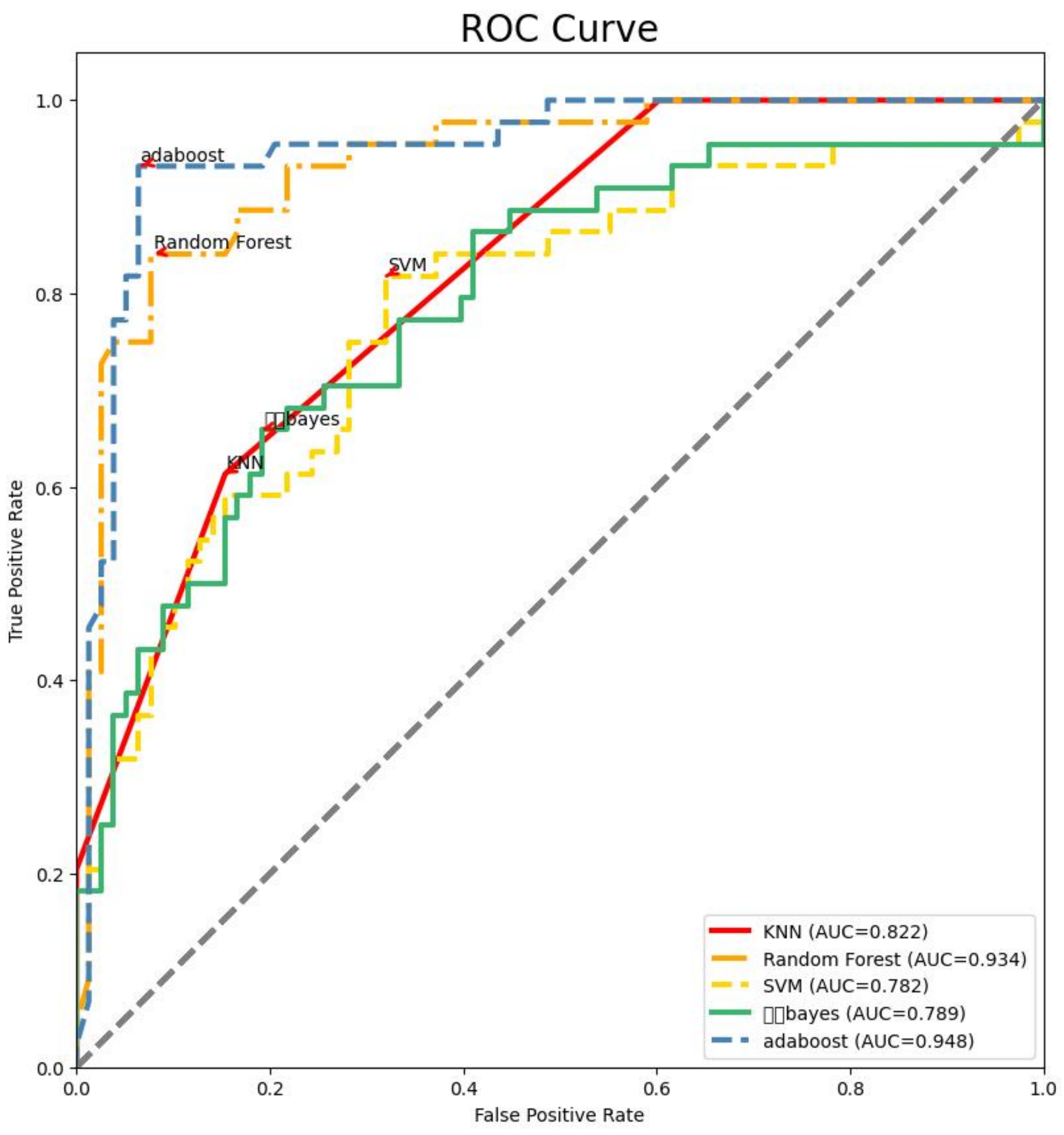

Figure S6 Results of experiment 3,K=5 (R5、R20、(R5-R20)/R5、AXV、Fres), CG vs PPFN, describing the diagnostic accuracy of Impulse oscillometry with ML algorithms in subjects with chronic respiratory symptoms and preserved pulmonary.

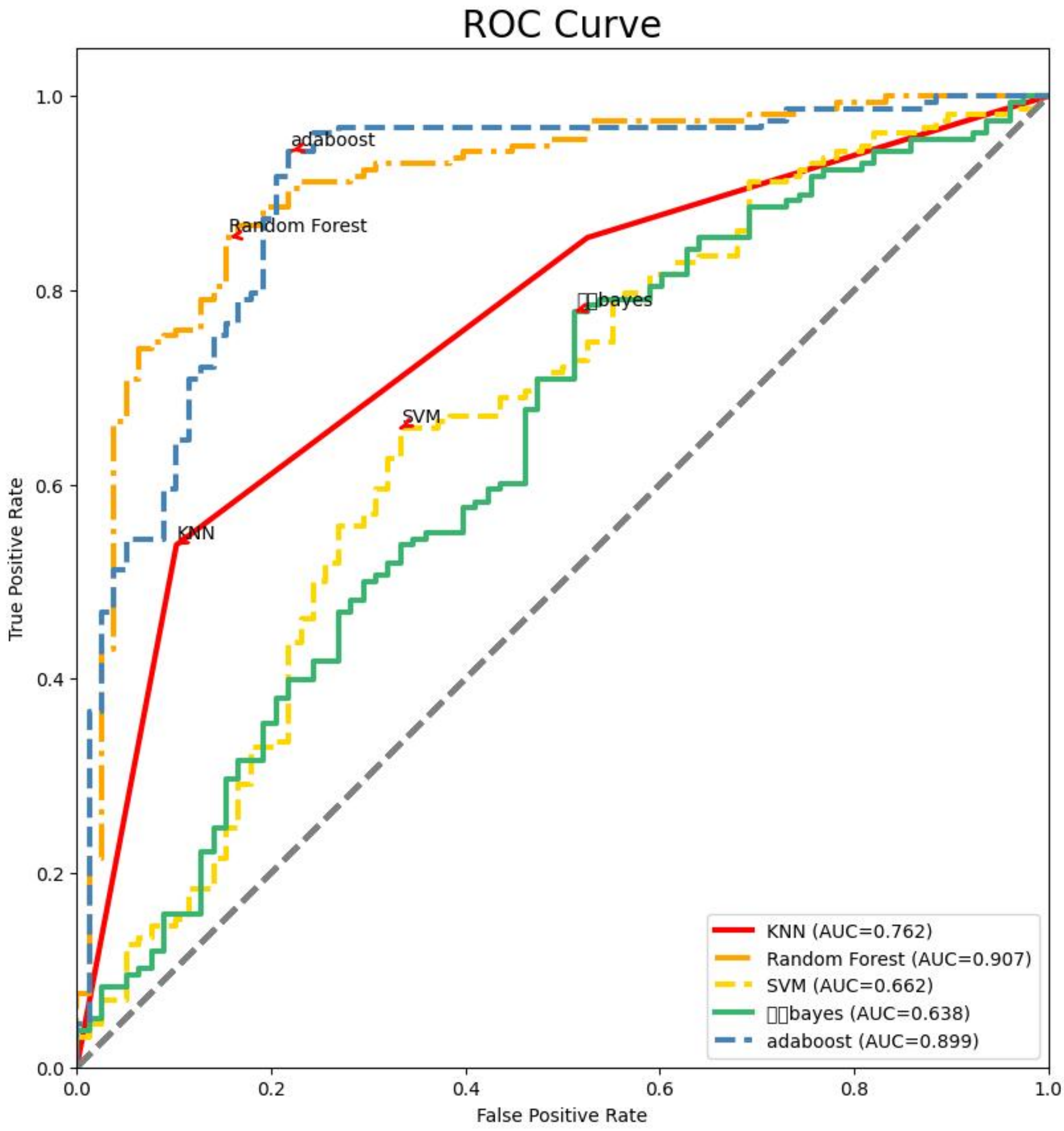

Figure S7 Results of experiment 3,K=5 (R5、R20 、(R5-R20)/R5、AXV、Fres), CG vs PPFA, describing the diagnostic accuracy of Impulse oscillometry with ML algorithms in subjects with chronic respiratory symptoms and preserved pulmonary.

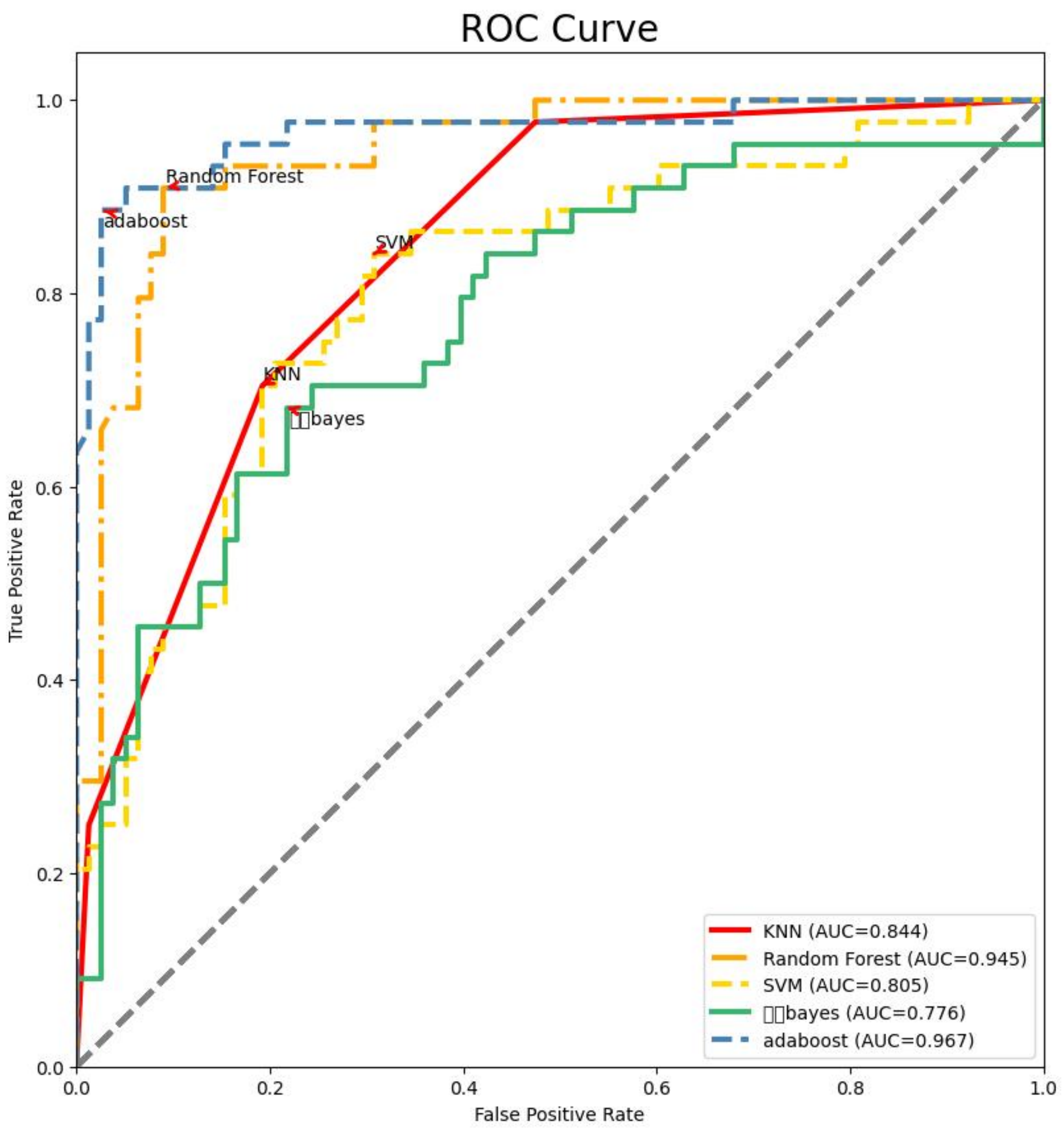

Figure S8 Results of experiment 4, RF as the model (R5、R20、(R5-R20)/R5、X5、AXV、Fres), CG vs PPFN, describing the diagnostic accuracy of Impulse oscillometry with ML algorithms in subjects with chronic respiratory symptoms and preserved pulmonary.

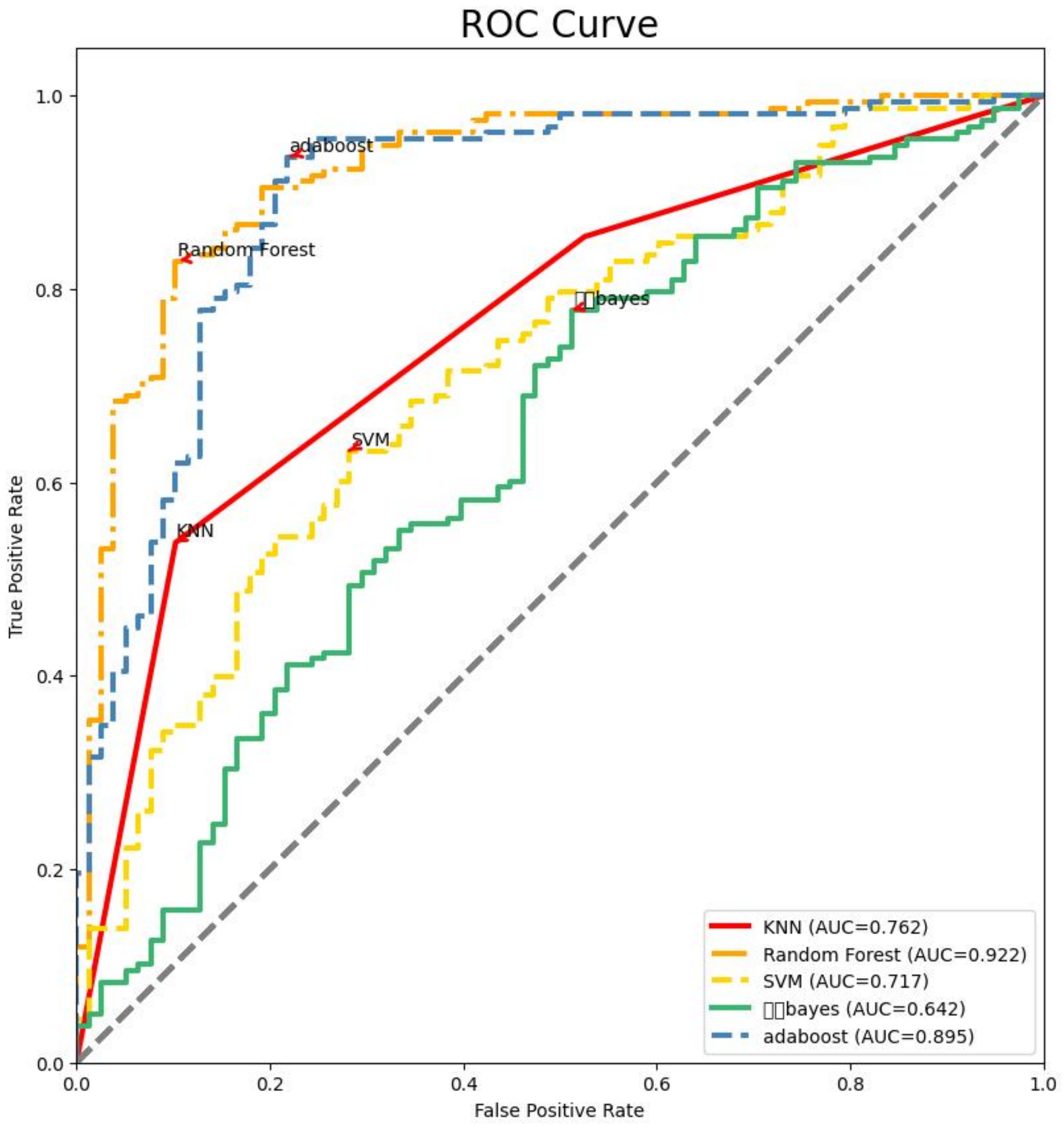

Figure S9 Results of experiment 4, SVM as the model (R5、R20、R5-R20、X5、AXV), CG vs PPFN, describing the diagnostic accuracy of Impulse oscillometry with ML algorithms in subjects with chronic respiratory symptoms and preserved pulmonary.

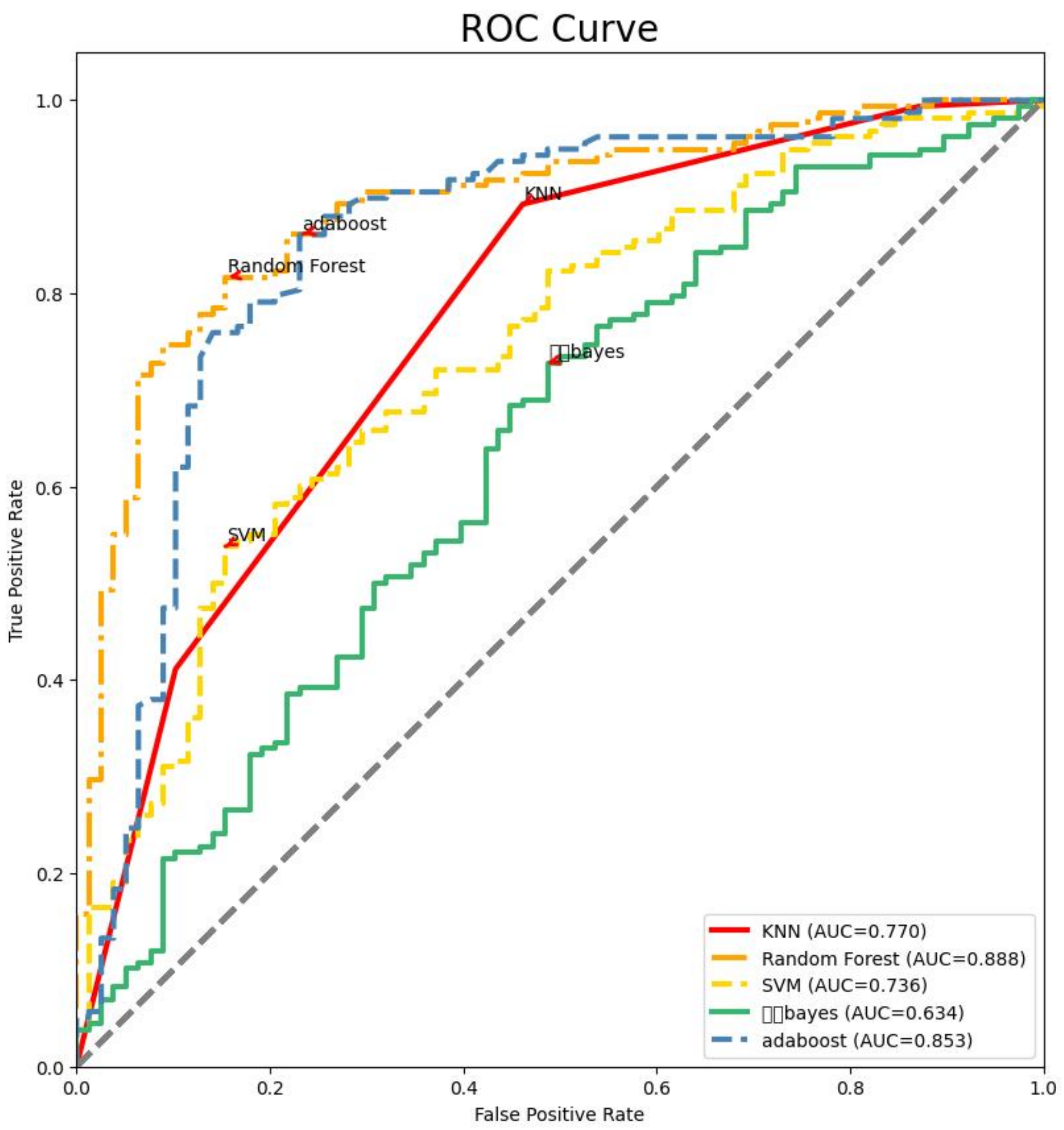

Figure S10 Results of experiment 4, ADABOOST as the model (Fres), CG vs PPFN, describing the diagnostic accuracy of Impulse oscillometry with ML algorithms in subjects with chronic respiratory symptoms and preserved pulmonary.

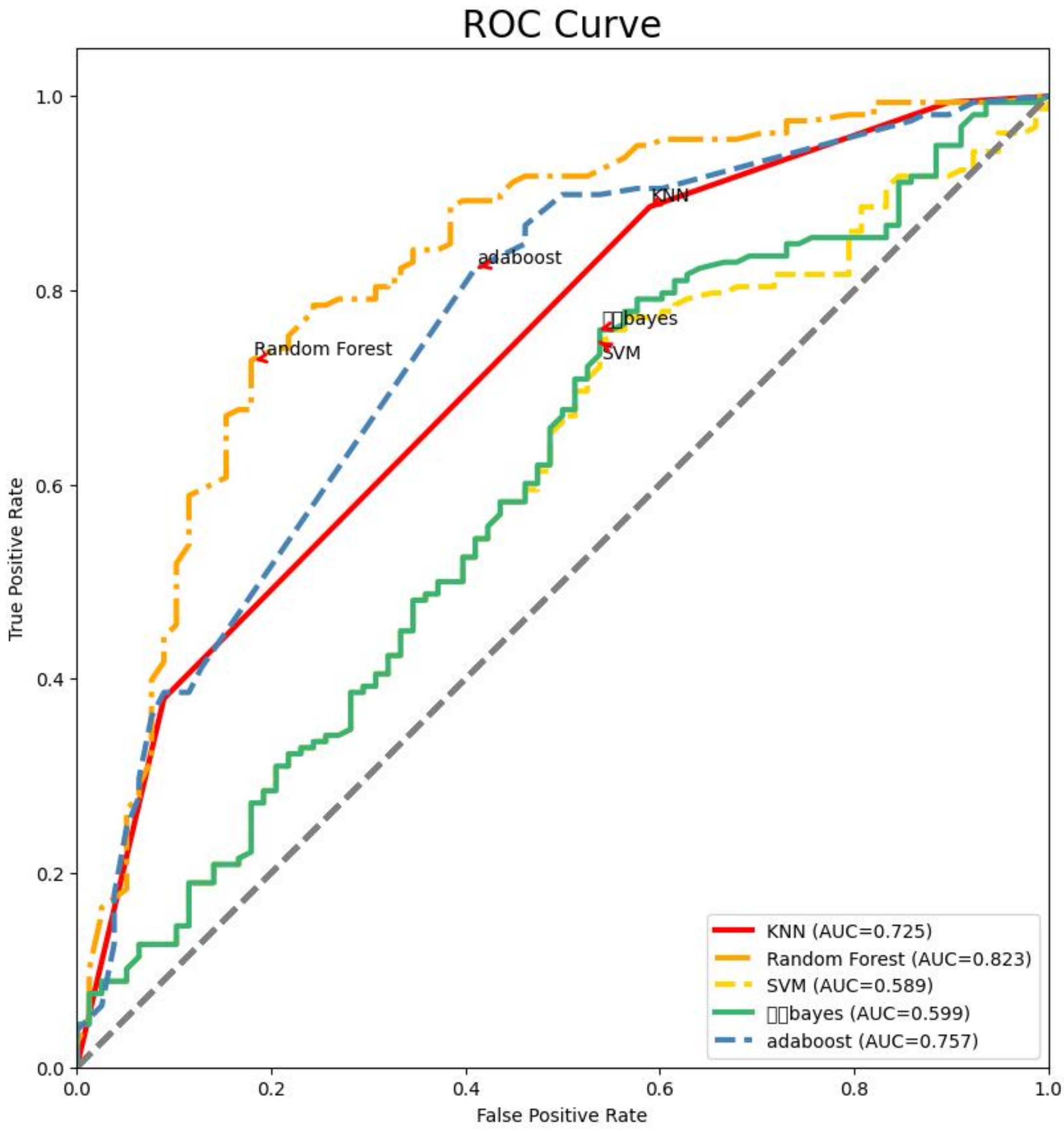

Figure S11 Results of experiment 4, RF as the model (R5、 Fres), CG vs PPFA, describing the diagnostic accuracy of Impulse oscillometry with ML algorithms in subjects with chronic respiratory symptoms and preserved pulmonary.

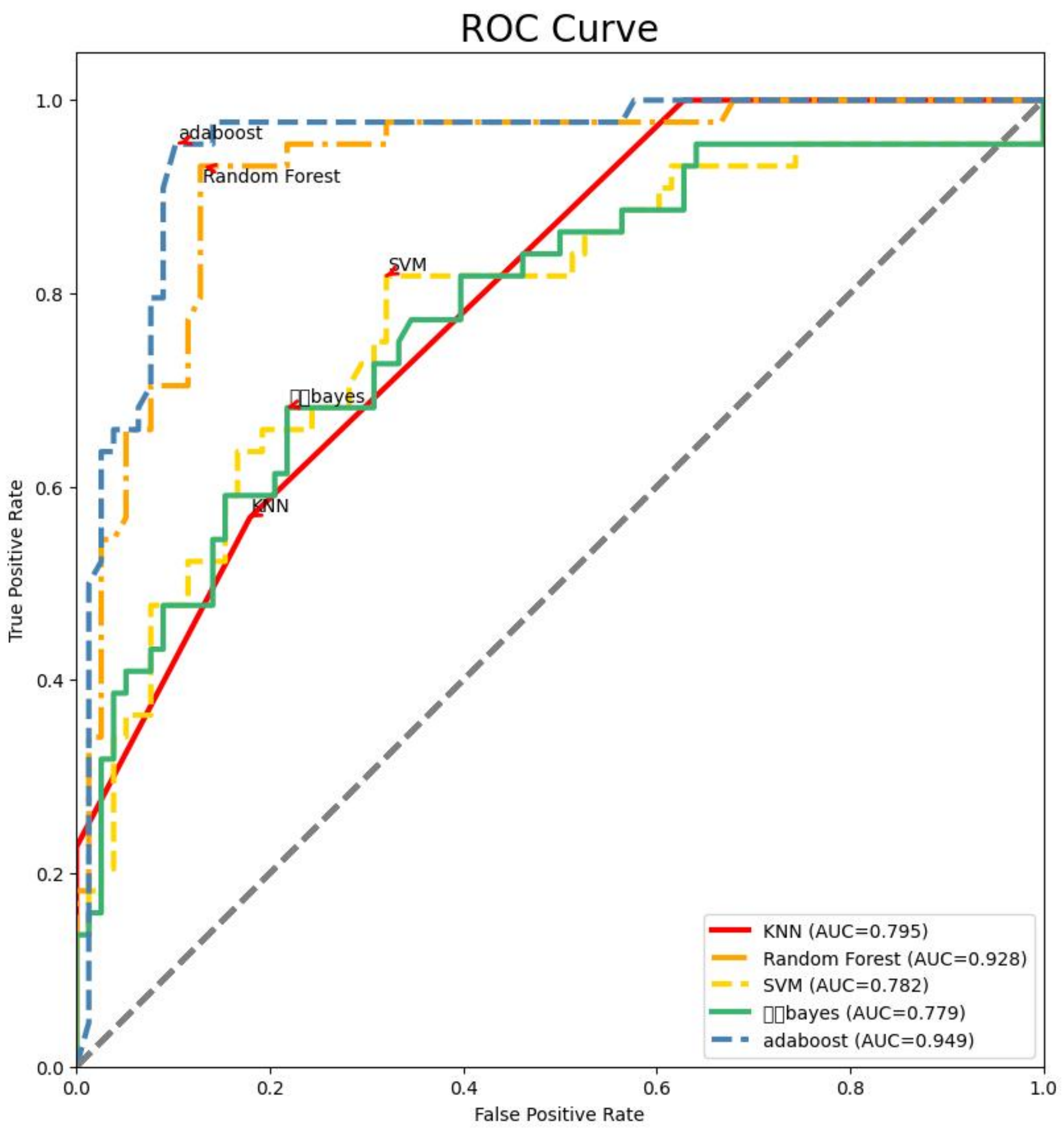

Figure S12 Results of experiment 4, SVM as the model (R5、R20、R5-R20、(R5-R20)/R5、X5、AXV), CG vs PPFA, describing the diagnostic accuracy of Impulse oscillometry with ML algorithms in subjects with chronic respiratory symptoms and preserved pulmonary.

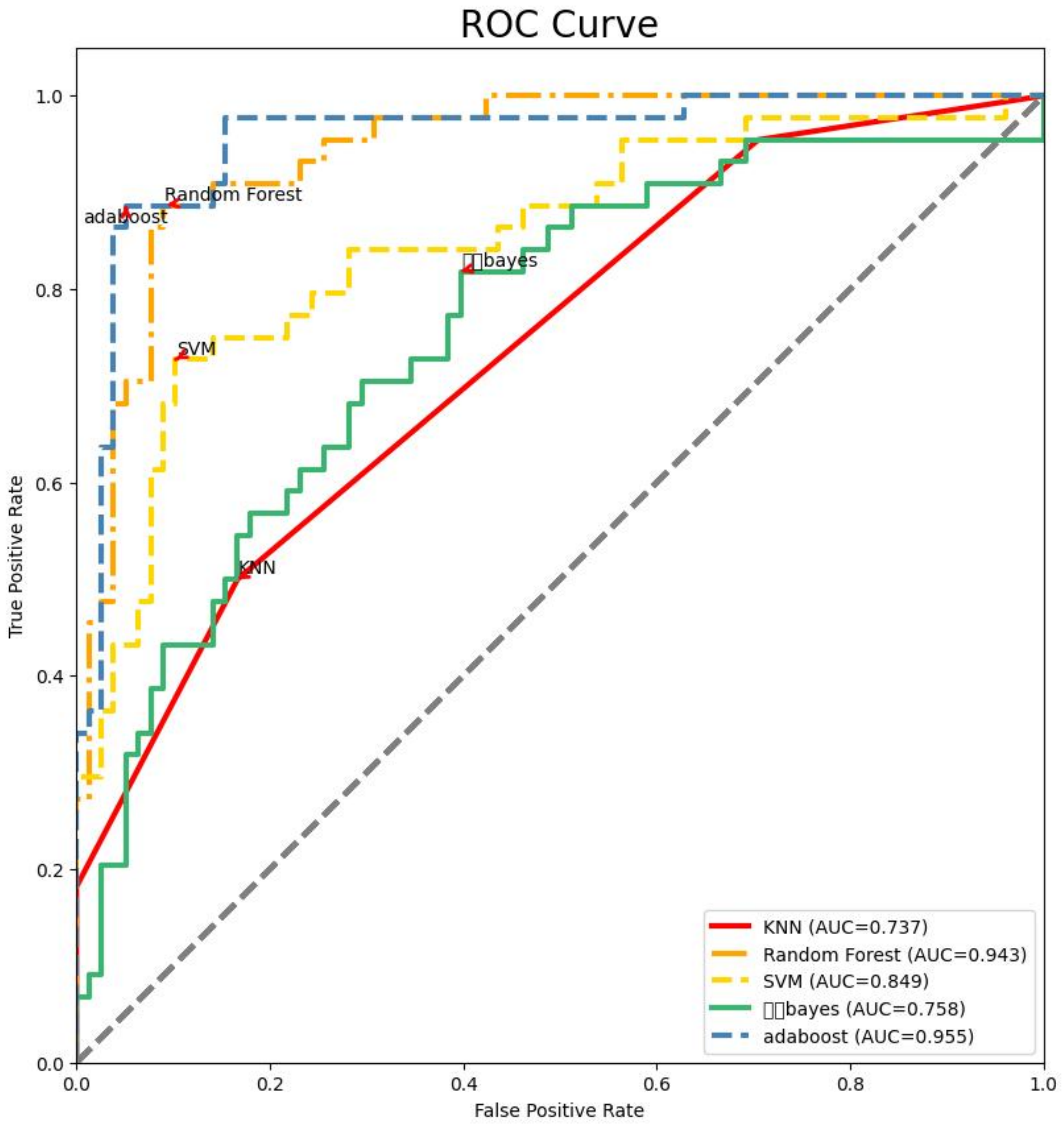

Figure S13 Results of experiment 4, ADABOOST as the model (R5、R20、(R5-R20)/R5、X5、AXV、Fres), CG vs PPFA, describing the diagnostic accuracy of Impulse oscillometry with ML algorithms in subjects with chronic respiratory symptoms and preserved pulmonary.

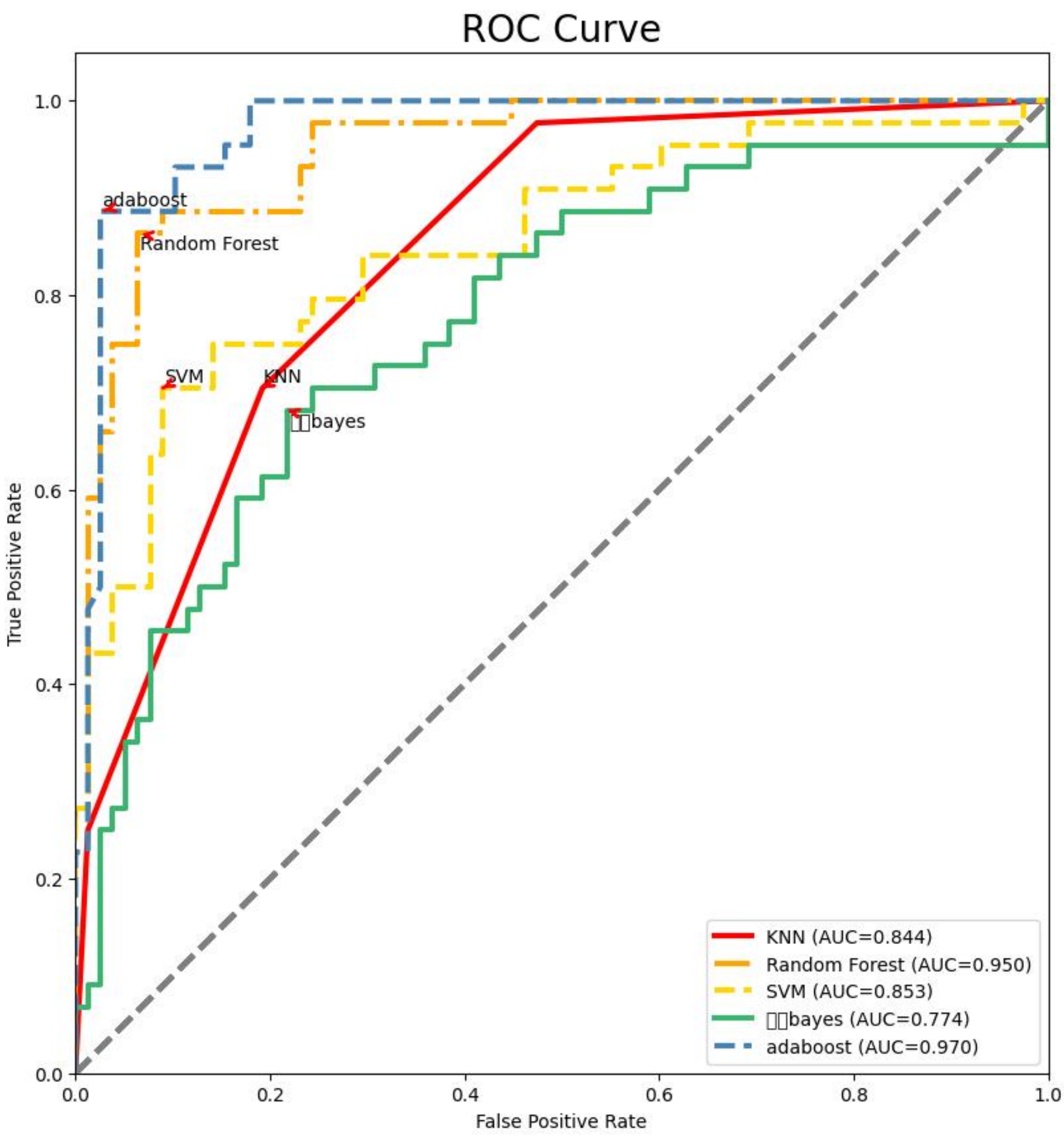

Figure S14 Results of experiment 5, RF as the model (R5、R20、(R5-R20)/R5、Fres), CG vs PPFN, describing the diagnostic accuracy of Impulse oscillometry with ML algorithms in subjects with chronic respiratory symptoms and preserved pulmonary.

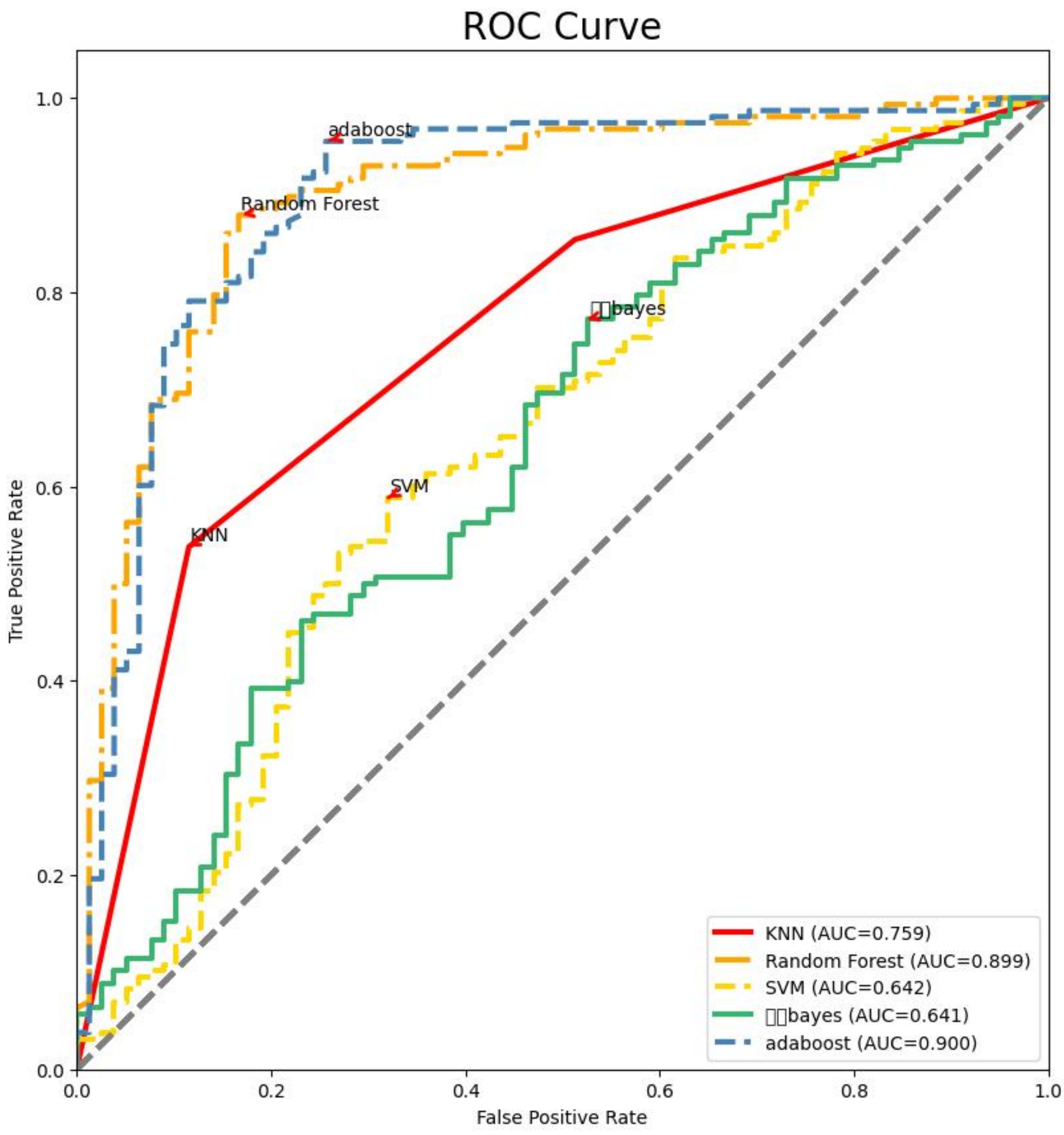

Figure S15 Results of experiment 5, SVM as the model (R5-R20、X5、AXV), CG vs PPFN, describing the diagnostic accuracy of Impulse oscillometry with ML algorithms in subjects with chronic respiratory symptoms and preserved pulmonary.

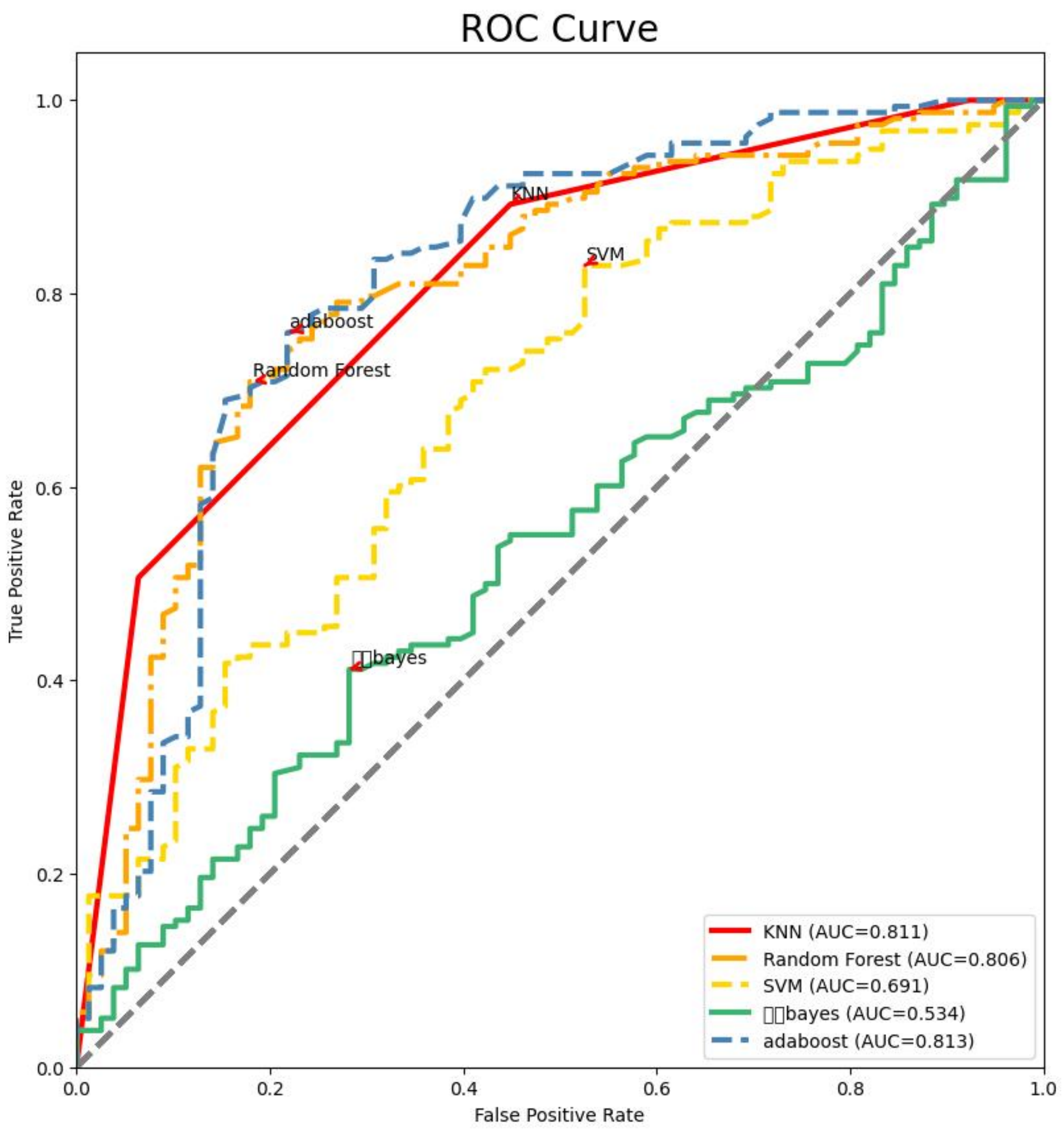

Figure S16 Results of experiment 5, ADABOOST as the model ((R5-R20)/R5、Fres), CG vs PPFN, describing the diagnostic accuracy of Impulse oscillometry with ML algorithms in subjects with chronic respiratory symptoms and preserved pulmonary.

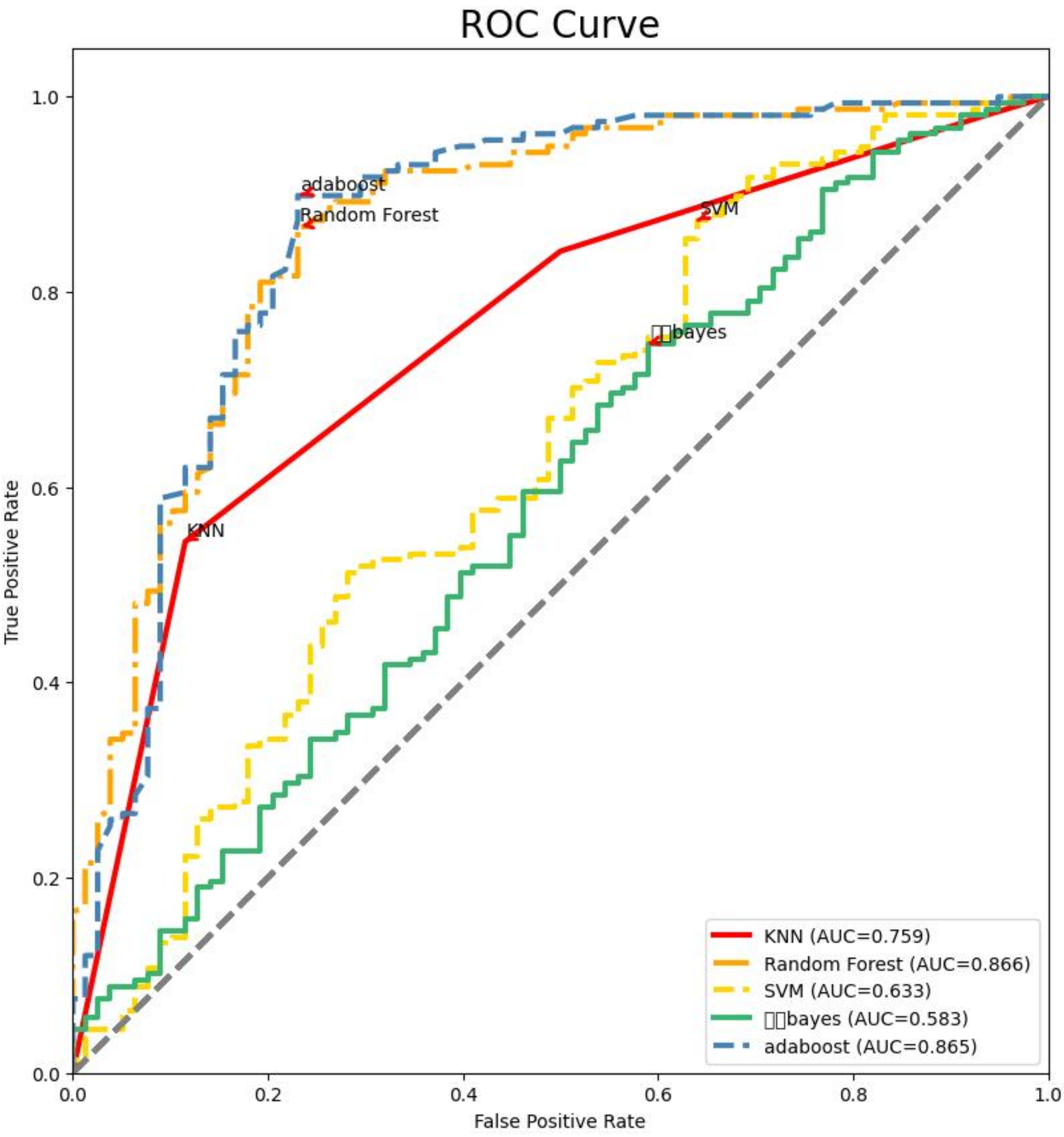

Figure S17 Results of experiment 5, RF as the model (R5、R20、Fres), CG vs PPFA, describing the diagnostic accuracy of Impulse oscillometry with ML algorithms in subjects with chronic respiratory symptoms and preserved pulmonary.

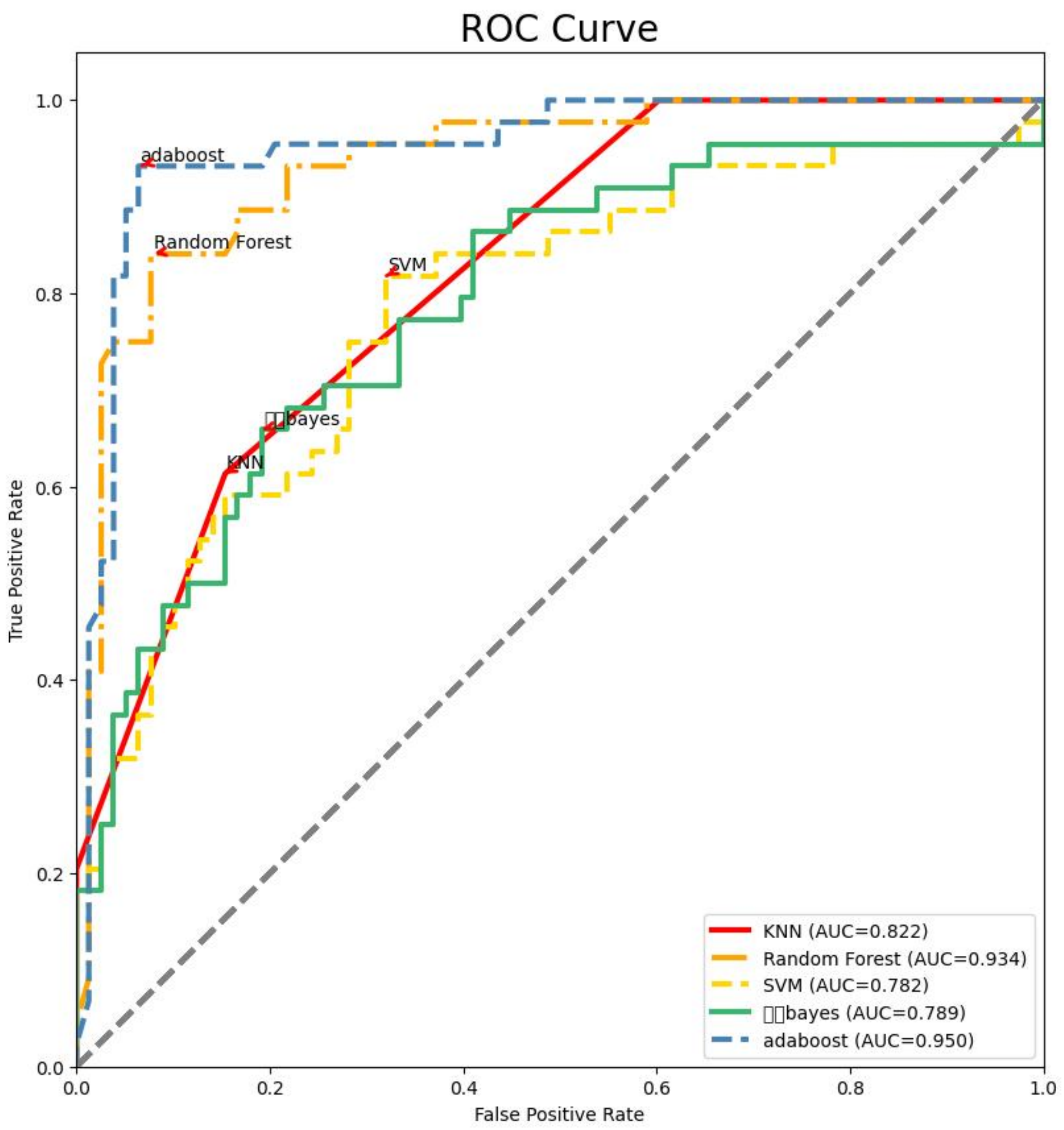

Figure S18 Results of experiment 5, SVM as the model (R5-R20、X5), CG vs PPFA, describing the diagnostic accuracy of Impulse oscillometry with ML algorithms in subjects with chronic respiratory symptoms and preserved pulmonary.

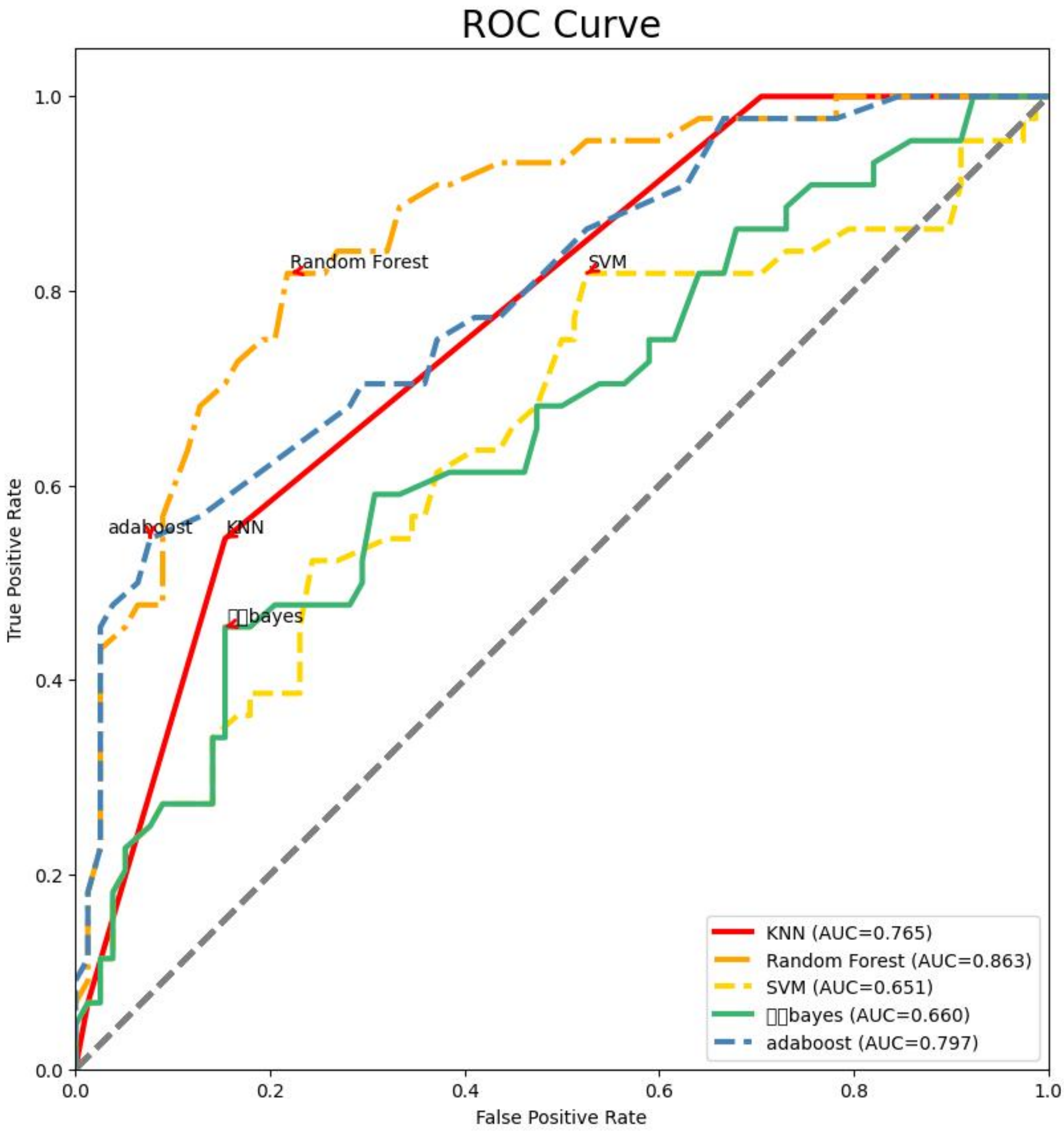

Figure S19 Results of experiment 5, ADABOOST as the model ((R5-R20)/R5、R5、Fres), CG vs PPFA, describing the diagnostic accuracy of Impulse oscillometry with ML algorithms in subjects with chronic respiratory symptoms and preserved pulmonary.

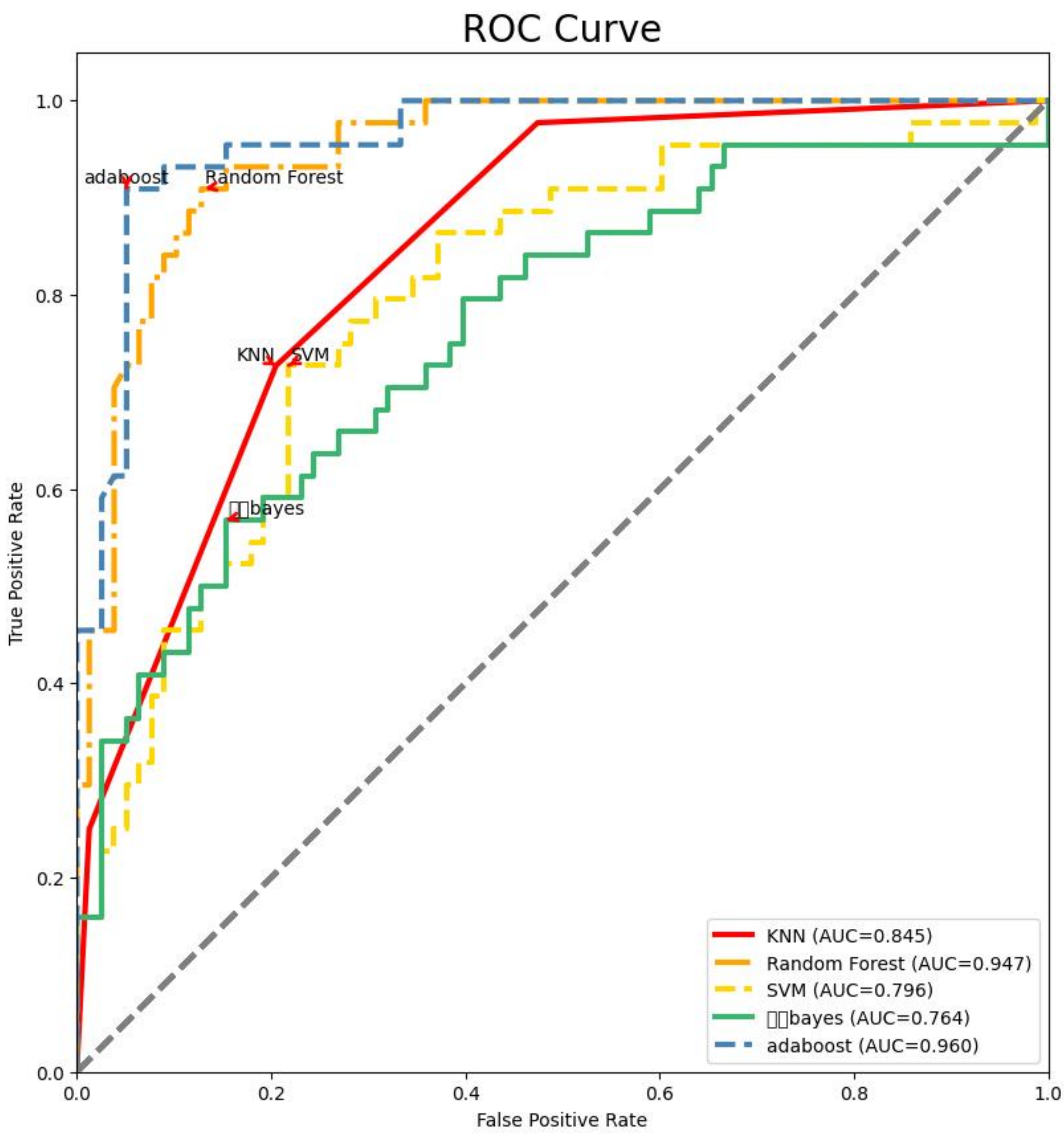

Supplement: Supplementary file 1 — Supplementary Material 1 [file 12931_2024_2911_MOESM1_ESM.pdf]
